# ﻿First record of subgenus *Synaldis* Foerster (Hymenoptera, Braconidae, Alysiinae, *Dinotrema* Foerster) from Chile, with description of ten new species

**DOI:** 10.3897/zookeys.1206.124515

**Published:** 2024-07-10

**Authors:** Franciélle Dias de Oliveira, Angélica Maria Penteado-Dias

**Affiliations:** 1 Programa de Pós-Graduação em Ecologia e Recursos Naturais, Universidade Federal de São Carlos, CP 676, CEP 13 565-905, São Carlos, SP, Brazil Universidade Federal de São Carlos São Carlos Brazil; 2 Universidade Federal de São Carlos, Departamento de Ecologia e Biologia Evolutiva, CP 676, CEP 13 565-905, São Carlos, SP, Brazil Universidade Federal de São Carlos São Carlos Brazil

**Keywords:** Alysiini, *Aspilota* group, endoparasitoid wasp, Ichneumonoidea, koinobiont, Neotropical region, parasitoid of Diptera, taxonomy

## Abstract

*Synaldis* is a taxon within the *Aspilota* group with a contentious taxonomic history, currently classified as a subgenus of the genus *Dinotrema*. Species of *Synaldis* were only documented in the Neotropical region in 2017, and until then, the Neotropical fauna of this subgenus was represented by five species from Brazil. In this study, *Synaldis* is reported for the first time in Chile, with the description and illustration of ten new species, namely: Dinotrema (Synaldis) acarinareolatum**sp. nov.**, D. (S.) brunneum**sp. nov.**, D. (S.) chilense**sp. nov.**, D. (S.) daltoni**sp. nov.**, D. (S.) flavum**sp. nov.**, D. (S.) latusdentertium**sp. nov.**, D. (S.) perisfelipoi**sp. nov.**, D. (S.) pilosicaudatum**sp. nov.**, D. (S.) puyehue**sp. nov.**, and D. (S.) verae**sp. nov.** The studied specimens were collected during expeditions to southern Chile, in the Valdivian temperate rainforest at Parque Nacional de Puyehue. This study also includes a dichotomous identification key for Neotropical species of *Synaldis*, as well as a discussion of the primary morphological characters used to distinguish species within the Neotropical and Nearctic regions.

## ﻿Introduction

The subfamily Alysiinae Leach, 1815 (Hymenoptera, Braconidae) contains koinobiont endoparasitoids exclusively of cyclorrhaphous Diptera larvae (Wharton 1984; van Achterberg 1993). Alysiinae is morphologically characterized by having exodont mandibles (outwardly directed, non-overlapping even when they are closed), and total loss of the occipital carina (van Achterberg 1993; Wharton 2017). This subfamily is subdivided into two tribes, Alysiini and Dacnusini, which differ by the presence of the fore wing vein r-m in Alysiini and its absence in Dacnusini (Shenefelt 1974; Yu et al. 2016).

Within Alysiini, the *Aspilota* group (sensu van Achterberg 1988) stands out as a remarkably large and complex group of genera. Members of this group are characterized by having a nearly glabrous apical portion of the ovipositor sheath, with its obtuse apex, and a host-spectrum nearly exclusively comprised of dipteran Phoridae. They are typically small, with a body length of 1–2 mm (less frequently ~ 3 mm), the body color is predominantly dark brown, and they are often found in decaying organic matter (van Achterberg 1988).

Two of the largest related genera in the *Aspilota* group, *Dinotrema* Foerster, 1863 and *Aspilota* Foerster, 1863 are morphologically distinguished by the size states of the paraclypeal fovea (anterior tentorial pit). In *Dinotrema*, this structure is small and clearly separated from the eye, whereas in *Aspilota*, the paraclypeal fovea is enlarged and almost reaching the margin of the eye (van Achterberg 1988). The genera *Dinotrema*, *Aspilota*, and related taxa are known for being among the most taxonomically complex within Braconidae. In addition to the predominantly small size of their representatives, the complexity is attributed to the limited characteristics used to distinguish species. Moreover, these diagnostic characters exhibit variability, sometimes significant, thereby obscuring the distinctions between closely related taxa (Belokobylskij and Tobias 2007).

Currently, the genus *Dinotrema* comprises three subgenera: the nominative *Dinotrema*, *Synaldis* Foerster, 1863, and *Synaldotrema* Belokobylskij & Tobias, 2002 (Zhu et al. 2017). *Synaldotrema* is distinguished by its anomalous metasomal structure, i.e., clearly narrowed towards the apex (in lateral view), with the apical sternites (and ovipositor) distinctly retracted under the long and protruding apical tergites, and fourth tergite very elongate (Belokobylskij and Tobias 2002, 2007). *Synaldis* differs from *Dinotrema* by the complete absence of vein 2-SR in the fore wing (consequently the first and second submarginal cells are confluents), while in the subgenus Dinotrema this vein is present, and the first and second submarginal cells are separated.

With a historically contentious taxonomic status, *Synaldis* was initially proposed as a genus by Foerster (1863). The generic validity of *Synaldis* has been questioned due to the variability in the reduction of certain veins among the Alysiini (Wharton 1980, 2002), including the 2-SR vein in specimens of the *Aspilota* group, as demonstrated by Koenig (1972). In 1988, van Achterberg re-established the genus *Dinotrema* and synonymized the species of “*Synaldis*” (having the paraclypeal fovea separated from the eye) with *Dinotrema*. Alternatively, *Synaldis* continued to be treated as a genus by several authors, and many species were either described in or transferred to it (Fischer 1993a, 1993b, 2003; Papp 1996, 2000; Belokobylskij 2002, 2004; Peris-Felipo et al. 2014a; Peris-Felipo and Belokobylskij 2017). Finally, Zhu et al. (2017) proposed recognizing *Synaldis* as a subgenus of *Dinotrema* for convenience, until a comprehensive phylogenetic study of the genus *Dinotrema* can support the recognition of *Synaldis* as a subgenus or genus, a classification that was employed in this study.

*Synaldis* has approximately 100 species described worldwide, and its members are often reared from agaric mushrooms and recorded as parasitoids of Phoridae and possibly Drosophilidae larvae (Peris-Felipo and Belokobylskij 2020). Peris-Felipo and Belokobylskij (2017) provided the initial record of *Synaldis* in the Neotropical region, along with an identification key for the previously known Nearctic and Neotropical species of the subgenus. Until then, five Neotropical species had been registered from Brazil: Dinotrema (Synaldis) brasiliense (Peris-Felipo, 2017), Dinotrema (Synaldis) fritzi (Peris-Felipo, 2017), Dinotrema (Synaldis) longiflagellaris (Peris-Felipo, 2017), Dinotrema (Synaldis) magnioculis (Peris-Felipo, 2017), and Dinotrema (Synaldis) novateutoniae (Peris-Felipo, 2017). In this study, we report the first record of *Synaldis* from Chile, with the description and illustration of ten new species: Dinotrema (Synaldis) acarinareolatum sp. nov., D. (S.) brunneum sp. nov., D. (S.) chilense sp. nov., D. (S.) daltoni sp. nov., D. (S.) flavum sp. nov., D. (S.) latusdentertium sp. nov., D. (S.) perisfelipoi sp. nov., D. (S.) pilosicaudatum sp. nov., D. (S.) puyehue sp. nov., D. (S.) verae sp. nov. Additionally, an identification key to the Neotropical species of *Synaldis* is provided.

## ﻿Materials and methods

**Figure 1.**
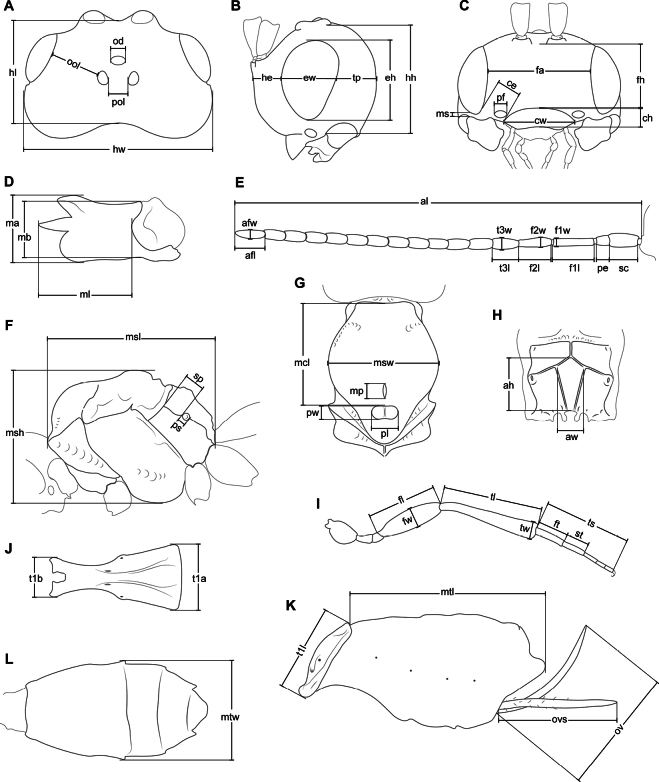
Measurements of head, mandible, antenna, mesosoma, leg, and metasoma **A, B, C** head, dorsal, lateral, and frontal view respectively **D** mandible, lateral view **E** antenna **F** mesosoma, lateral view **G** mesonotum, dorsal view **H** propodeum, dorsal view **I** hind leg **J** first metasomal tergite (T1), dorsal view **K** metasoma, lateral view **L** metasoma (without T1), dorsal view. Abbreviations: afl – apical flagellar segment length, afw – apical flagellar segment width, ah – areola height, al – antenna length, aw – areola width, ce – distance from clypeus to eye, ch – clypeus height, cw – clypeus width, eh – eye height, ew – eye width, f1l – first flagellar segment length, f1w – first flagellar segment width, f2l – second flagellar segment length, f2w – second flagellar segment width, fa – face width, fh – face height, fl – femur length, ft – first segment of tarsus (basitarsus) length, fw – femur width, he – head (partial) extension, hh – head height, hl – head length, hw – head width, ma –mandibular apical width, mb –mandibular basal width, mcl – mesoscutum length, ml – mandible length, mp – mesoscutal pit length, ms – malar space, msh – mesosoma height, msl – mesosoma length, msw – mesosoma width, mtl – metasoma (partial) length, mtw – metasoma width, od – ocellus diameter, ool – ocular-ocellar line, ov – ovipositor length, ovs – ovipositor sheath length, pe – pedicel length, pf – paraclypeal fovea diameter, pl – prescutellar depression length, pol – posterior-ocellar line, ps – propodeal spiracle diameter, pw – prescutellar depression width, sc – scape length, sp – distance from spiracle to base of propodeum, st – second segment of tarsus length, t1a – first metasomal tergite apical width, t1b – first metasomal tergite basal width, t1l – first metasomal tergite length, t3l – third flagellar segment length, t3w – third flagellar segment width, tl – tibia length, tp – temple width, ts – tarsus length, tw – tibia width.

The nomenclature of wing venation follows van Achterberg (1993), and body sculpture follows Eady (1968). The other morphological terms and measurements were based on Peris-Felipo et al. (2014b), with additional explanations provided below. Body length: in lateral view, sum of the head extension (Fig. 1B, he+ew+tp), mesosoma length (Fig. 1F, msl), and metasoma length (Fig. 1K, t1l+mtl). In dorsal view, head width is its maximum width (at eyes or temples), and head length is the midline between frons anteriorly and occiput. For head measurements in lateral view (Fig. 1B), the head was positioned to vertically align the upper base of the mandible with the lateral ocellus (following Wharton 1977). Paraclypeal fovea size: ratio between the maximum diameter of the fovea and the shortest distance from the fovea to the eye (short ≤ 0.40, middle = 0.45–0.55) (Fig. 1C). The mandible width is its maximum width (at apex or base) (Fig. 1D); diagonal carina refers to a carina arising from upper tooth (Fig. 9). Antenna length: sum of the lengths of its segments (Fig. 1E). The width of the first flagellar segment (F1) is its apical width, while for the other flagellomeres the width is their maximum width. Maxillary palp length: sum of the lengths of its segments.

Mesosoma width is the maximum width of mesoscutum; prescutellar depression (scutellar sulcus) width is its maximum width (Fig. 1G). Propodeal areola height and width were measured inside the areola (Fig. 1H). Propodeal spiracle size: ratio between the diameter of spiracle (at its outer margin) and the shortest distance from the spiracle to the basal margin of propodeum (small ≤ 0.3, middle = 0.35–0.50, large ≥ 0.55), in lateral view (Fig. 1F). Hind femur width is its maximum width and hind tibia width is its subapical width (Fig. 1I). Hind tarsus length: sum of the lengths of its segments. Metasoma length: sum of the first metasomal tergite (T1) length and the distance from anterior margin of the second tergite to the metasomal apex, in lateral view (Fig. 1K; t1l+mtl). Metasoma width is its maximum width in dorsal view (Fig. 1L).

**Figure 2.**
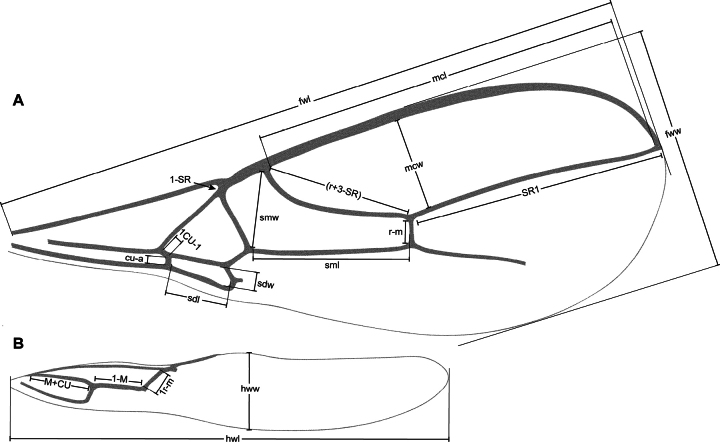
Wing measurements **A** fore wing **B** hind wing. Abbreviations: fwl – fore wing length, fww – fore wing width, hwl – hind wing length, hww – hind wing width, mcl – marginal cell length, mcw – marginal cell width, sdl – subdiscal cell length, sdw – subdiscal cell width, sml – submarginal cell length, smw – submarginal cell width.

The wing veins and cells mentioned in the descriptions and identification key, along with their respective measurements, are depicted in Fig. 2. For these wing veins, the corresponding terminologies from Fischer (1972) and Wharton et al. (2017) are respectively provided, in parentheses, as follows: fore wing – 2-SR (cuqu1, 2SR); r (r1, r); 3-SR (r2, 3RSa); SR1 (r3, 3RSb); cu-a (nv, 1cu-a); hind wing – m-cu (n. rec., m-cu). Additionally, the equivalent terminology of Fischer (1972) for the wing cells is: marginal (radial) cell, first + second submarginal (cubital) cells, and first subdiscal (brachial) cell. The width of the wings corresponds to their maximum width. In the fore wing, the term submarginal cell refers to the first + second submarginal cells. The length of vein (r+3-SR) was measured as the straight-line distance between its intersection with the pterostigma and the r-m vein (Fig. 2A).

**Figure 3.**
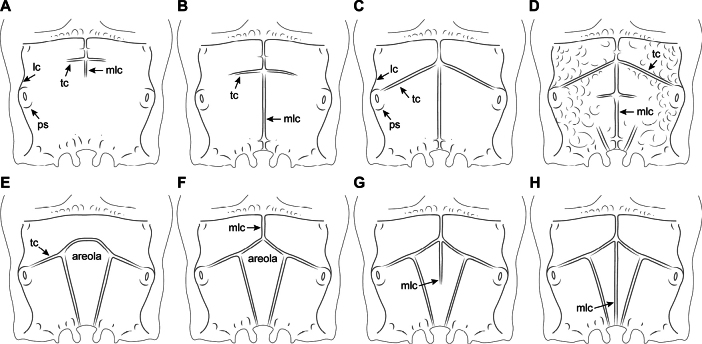
Schematic representation of the various types of propodeal sculpture and areolation in New World species of *Synaldis***A** propodeum with median longitudinal carina and transverse carinae incomplete, short **B** propodeum with median longitudinal carina complete and transverse carinae incomplete **C** propodeum mainly smooth, with median longitudinal carina and transverse carinae complete **D** propodeum mainly rugose, with median longitudinal carina and transverse carinae complete (areola absent) **E** propodeum with areola and transverse carinae complete (median longitudinal carina absent) **F** propodeum with areola and transverse carinae complete, median longitudinal carina incomplete, basal (not extending inside the areola) **G** propodeum with areola and transverse carinae complete, median longitudinal carina incomplete apically, reaching mid-areola **H** propodeum with areola, median longitudinal carina and transverse carinae complete. Abbreviations: lc – lateral carina, mlc – median longitudinal carina, ps – propodeal spiracle, tc – transverse carina.

Different types of propodeal sculpture and areolation are schematically represented in Fig. 3. The propodeal median longitudinal carina was considered incomplete when it is clearly interrupted (Fig. 3A, F, G), and complete when it crosses the propodeum from the basal to apical margin (Fig. 3B–D, H). Transverse carinae are incomplete when distinctly separated from the lateral parts (sides) of propodeum (Fig. 3A, B), and complete when they extend to the lateral of propodeum, at spiracle margin or lateral carina (Fig. 3C–H). The propodeal surface and the development of carinae were evaluated independently. For instance, the propodeum may exhibit a mainly smooth surface combined with poorly developed carinae (Fig. 3A), or a mainly smooth surface along with a distinct areola and complete carinae (Fig. 3H). A widely sculptured propodeum may exhibit distinct carination (as depicted in Fig. 3D), or the carinae may be lacking or indiscernible.

Digital scanning electronic microscope (SEM) photographs of uncoated specimens were taken with a FEI Quanta 250 SEM in a low vacuum mode. Color digital photographs were taken with a Leica M250C stereomicroscope, using a Leica MC170 HD camera and Leica Application Suite software v. 4.12. Measurements of the specimens were conducted using digital photographs taken with a Leica M165C stereomicroscope, Leica DFC295 HD camera, and Leica Application Suite software v. 3.7. Adobe Illustrator v. 24.1.2 was utilized for illustrations, and Adobe Photoshop CS5 Extended v. 12.1. for minor adjustments to photographs and preparation of the plates.

Abbreviations used throughout the descriptions are as follows: **POL** post-ocellar line (shortest distance between lateral ocelli), **OD** ocellus diameter (maximum diameter of ocellus), **OOL** ocular-ocellar line (shortest distance between lateral ocellus and eye), **F1** first flagellar segment, **F2** second flagellar segment, **F3** third flagellar segment, **AF** apical flagellar segment, **T1** first metasomal tergite.

Type specimens were collected by Dr D. S. Amorim and Dr V. C. Silva, with loans provided by the former. They originate from collections conducted during expeditions to the southern Chile in Valdivian temperate rainforest at Parque Nacional Puyehue (refer to Amorim et al. 2022). The holotypes and some paratypes are deposited in the Entomology Area of the Museo Nacional de Historia Natural, Santiago, Chile (**MNNC**), while the remaining paratypes are deposited in the Coleção Taxonômica do Departamento de Ecologia e Biologia Evolutiva, São Carlos, Brazil (**DCBU**).

## ﻿Taxonomic account

**Subfamily Alysiinae Leach, 1815**

**Tribe Alysiini Leach, 1815**

### ﻿Genus Dinotrema

Taxon classificationAnimaliaHymenopteraBraconidae

Foerster, 1863

027B307B-7BD8-50D5-90D2-3250D4730FE0

#### Type species.

*Dinotremaerythropa* Foerster, 1863.

### ﻿ Subgenus Synaldis

Taxon classificationAnimaliaHymenopteraBraconidae

Foerster, 1863

8792A826-CB7B-5EB5-9627-6BD3507716B8

#### Type species.

*Bassusconcolor* Nees von Esenbeck, 1812 (monobasic).

Foerster 1863: 273 (original designation as genus); van Achterberg 1988: 21 (as synonym of *Dinotrema*); Fischer 1993a: 452 (as genus); Zhu et al. 2017: 38 (as subgenus).

#### Diagnosis.

Mandibles tridentate, teeth of differing shape and length, sometimes upper tooth very small. Paraclypeal fovea small, clearly separated from eye. Precoxal sulcus always present. Pterostigma very long and narrow. Fore wing vein 2-SR always absent, resulting the first and second submarginal cells confluent; break between veins r and 3-SR absent. Vein cu-a often postfurcal, rarely almost interstitial. Metasoma with tergites not very narrowed apically in lateral view, apical sternites and ovipositor not strongly retracted under long apical tergites.

#### Hosts.

Diptera larvae of the family Phoridae and possibly Drosophilidae.

#### Comments.

The subgenus *Synaldis* Foerster, 1863 from the genus *Dinotrema* is recorded in the fauna of Chile for the first time.

### ﻿ Dinotrema (Synaldis) acarinareolatum sp. nov.

Taxon classificationAnimaliaHymenopteraBraconidae

EBA9BE8F-C155-5B92-8099-76E57A88CA0B

https://zoobank.org/EB2DFDFC-9722-46DB-8CBE-C8C067947E3E

Figs 4–12

#### Type material.

***Holotype***: Chile • ♀ (MNNC); Osorno, Parque Nacional Puyehue, Antillanca; 40°46'55"S, 72°12'39"W; alt. 987 m; 9–23 Dec. 2019; D. Amorim and V. Silva leg.; Malaise trap. ***Paratypes***: Chile • 1♀ (DCBU 514718) and 1♂ (MNNC); same data as for holotype.

#### Diagnosis.

**Figures 4–12.**
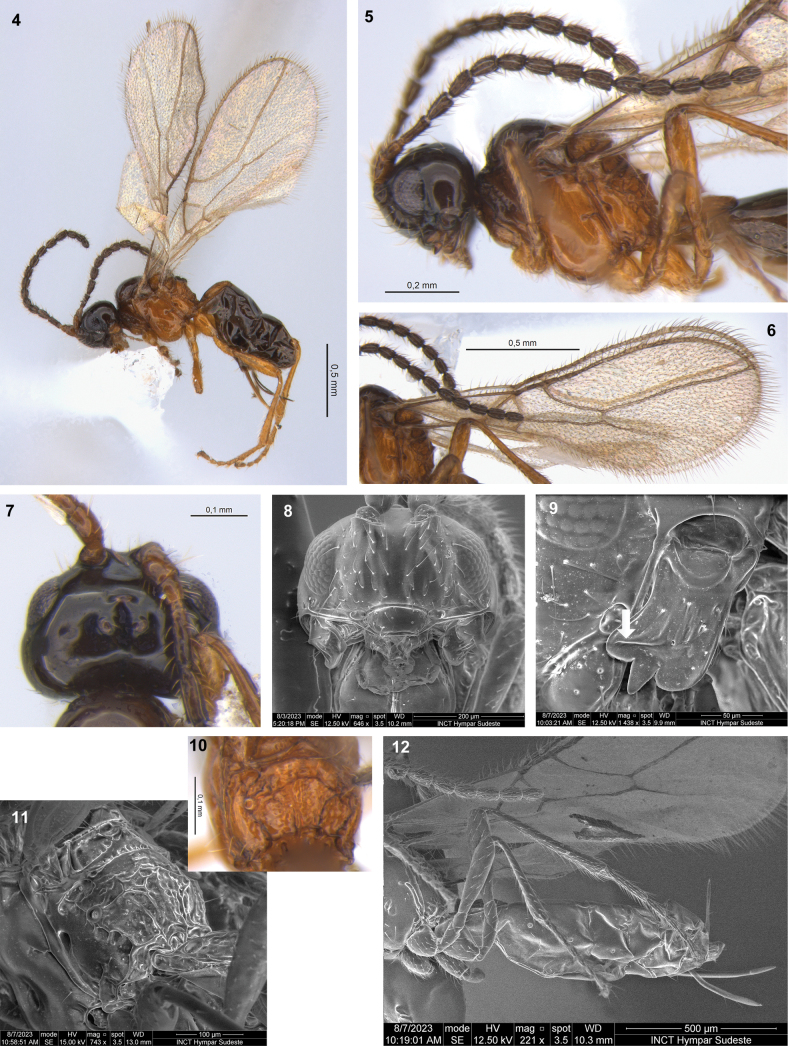
Dinotrema (Synaldis) acarinareolatum sp. nov. (**4**, **10** holotype ♀, remainder paratypes ♀, except **7**, **11** ♂) **4** habitus, lateral view **5** antenna, head and mesosoma, lateral view **6** fore wing **7** head, dorsal view **8** head, frontal view **9** mandible, lateral view, arrow showing the diagonal carina **10, 11** propodeum, dorsal and laterodorsal view **12** hind leg, metasoma and ovipositor, lateral view.

This species differs from other New World species of *Synaldis* by the sculpture of the propodeum, with distinct areola and transverse carinae complete, but median longitudinal carina absent (Figs 3E, 10, 11). Additionally, D. (S.) acarinareolatum sp. nov. can be differentiated by combination of following characteristics: OOL of ♀ 4.0× as OD (Fig. 7); in lateral view, eye shorter than temple (Fig. 5); mandible with three relatively large teeth, diagonal carina distinct, mandibular apex wider than base (Fig. 9); F1 2.4–2.5× as long as wide (Fig. 5); mesoscutal pit present, but sometimes very weak; fore wing vein cu-a postfurcal, 1-CU1 slightly shorter than cu-a, first subdiscal cell 2.5× as long as wide (Figs 4, 6), hind wing 6.2× as long as wide, vein 1-M 2.3–2.4× as long as 1r-m; hind tibia 8.5–8.7× as long as wide.

#### Description.

♀. Length. Body: 1.8–1.9 mm. Fore wing: 1.6–1.8 mm. Hind wing: 1.2–1.3 mm.

***Head***: in dorsal view (Fig. 7), 1.50–1.65× as wide as long, 1.5–1.6× as wide as mesosoma, wider at temples than eyes. Frons smooth. POL 1.5× as OD, OOL 4.0× as OD. In lateral view (Fig. 5), eye 1.4–1.5× as high as wide, 0.6–0.7× as wide as temple. Face 1.8× as wide as high (Fig. 8), 1.8× as wide as clypeus, with a weak longitudinal ridge dorsally. Clypeus 2.4× as wide as high, concave ventrally. Malar space 0.7× as clypeus height. Paraclypeal fovea short. Mandible 1.2–1.3× as long as wide (Fig. 9), diagonal carina present, strong. Mandibular apex 1.2× wide as base. Upper tooth rounded. Middle tooth subacuminate, longer than other teeth. Lower tooth largely rounded, as long as upper tooth. Lower tooth slightly wider than upper, both wider than middle tooth. Antenna with 15–16 segments (Fig. 5), 0.7–0.8× as long as body. Scape 1.7× as long as pedicel. F1 2.4–2.5× as long as wide, 1.1–1.2× as long as F2. F2 1.6–1.8× as long as wide. F3 1.4–1.7× as long as wide. AF 1.8× as long as wide. Maxillary palp 0.8× as long as head height.

***Mesosoma***: 1.2–1.3× as long as high (Fig. 5), 2.0–2.1× as long as wide. Mesoscutum approximately as long as wide, notauli absent on horizontal surface of mesoscutum. Mesoscutal pit present, distinct and oval, or very weak and rounded, occupying 0.1× of mesoscutal length. Prescutellar depression 1.9–2.1× as long as wide, with median carina incomplete anteriorly to complete, smooth laterally. Side of pronotum weakly crenulate. Precoxal sulcus crenulate medially, not reaching anterior and/or posterior margins of mesopleuron. Posterior mesopleural furrow smooth. Propodeum mainly rugulose to rugose (Figs 10, 11), with areola 0.9× as high as wide; median longitudinal carina absent; transverse carinae complete. Propodeum with very weak protuberance in lateral view. Propodeal spiracle small (Fig. 5), 0.3× distance from spiracle to base of propodeum.

***Wings***: fore wing 2.7× as long as wide, vein 1-SR present, (r+3-SR) 5.7–5.8× as long as r-m, SR1 2.40–2.55× as long as (r+3-SR); cu-a postfurcal, 1-CU1 0.9× as long as cu-a. Marginal cell 4.00–4.25× as long as wide, submarginal cell 2.0–2.2× as long as wide, first subdiscal cell 2.5× as long as wide (Figs 4, 6). Hind wing 6.2× as long as wide, vein 1-M 0.6–0.7× as long as M+CU, 2.3–2.4× as long as 1r-m; m-cu absent.

***Legs***: hind femur 4.0× as long as wide. Hind tibia 8.5–8.6× as long as wide, 1.1× as long as hind tarsus. First segment of hind tarsus 2.0–2.1× as long as second segment (Fig. 12).

***Metasoma***: 1.7–1.9× as long, and 1.3× as wide as mesosoma. T1 strigose, 1.9× as long as wide, apex 1.4× as wide as base. Ovipositor 0.4× as long as metasoma, 1.5–2.0× as long as T1, 1.1–1.3× as long as hind femur. Ovipositor sheath with some sparse and delicate setae (except on 1/3 apical almost glabrous), 0.3–0.4× as long as metasoma, 1.4× as long as T1 (Figs 4, 12).

***Color***: head, antennae, pronotum, mesoscutum and metasoma from the second tergite dark brown to brown. Mandibles and side of pronotum light brown. Remaining parts of mesosoma, legs, T1, and ovipositor yellowish. Wings hyaline, veins brown.

**Male.** Body length 1.6 mm. POL 1.3× OD, OOL 3.0× OD. Face 1.7× as wide as high, 2.1× as wide as clypeus. Clypeus 2.0× as wide as high. Mandible 1.4× as long as wide. Antenna with 18 segments, as long as body. F1 as long as F2. F2 2.0× as long as wide. F3 1.9× as long as wide. Maxillary palp as long as head height. Mesosoma 2.2× as long as wide. Prescutellar depression 1.8× as long as wide. Hind femur 4.2× as long as wide. Hind tibia 8.7× as long as wide. Metasoma 1.5× as long as mesosoma.

#### Etymology.

The epithet is an adjective combining *acarina* (prefix *a*- indicating negation, with *carina* from Latin) and *areolatum* (derived from *areola* in Latin). The species name refers to the sculpture of propodeum, which lacks a median longitudinal carina and has a distinct areola (Figs 10, 11).

#### Distribution.

Chile.

#### Comments.

Based on its eye being shorter than temple, as well as its relatively thickened flagellomeres and legs, D. (S.) acarinareolatum sp. nov. appears to be related to the described here D. (S.) daltoni sp. nov., D. (S.) perisfelipoi sp. nov. and D. (S.) puyehue sp. nov., especially to the former. The differences between these species are given in the identification key.

### ﻿ Dinotrema (Synaldis) brunneum sp. nov.

Taxon classificationAnimaliaHymenopteraBraconidae

359EC3AD-C2B1-585C-A5D1-9AB97CC3472E

https://zoobank.org/05E15272-7217-4B01-9F5C-90BA7275B75C

Figs 13–22

#### Type material.

***Holotype***: Chile • ♀ (MNNC); Osorno, Parque Nacional Puyehue, Antillanca; 40°44'06"S, 72°19'47"W; alt. 528 m; 14 Jan.–3 Feb. 2017; D. Amorim and V. Silva leg.; flight intercept trap. ***Paratypes***: Chile • 1♂ (MNNC); same data as for holotype, except 40°44’S, 72°19’W; alt. 440 m; sweeping • 2♀♀ (DCBU 387261, DCBU 387295); same data as for holotype, except 40°44’S, 72°19’W; alt. 440 m; sweeping.

#### Diagnosis.

This species differs from other New World species of *Synaldis* by the combination of the following characteristics: in lateral view, eye wider than temple (Fig. 17); paraclypeal fovea middle size (Fig. 14); mandible with three relatively large teeth, diagonal carina weak, mandibular apex wider than base; F1 2.7–3.3× as long as wide (Fig. 15); mesoscutal pit present, conspicuous (Fig. 16); propodeum with areola, median longitudinal carina and transverse carinae complete (Fig. 21); fore wing vein cu-a postfurcal, 1-CU1 shorter than cu-a (Fig. 18); hind tibia 9.8–10.3× as long as wide (Fig. 20).

Dinotrema (S.) brunneum sp. nov. is similar to D. (S.) chilense sp. nov. (see their differences in the identification key) and D. (S.) verae sp. nov., from which it can be distinguished by head and mesoscutum brown to dark brown (head dorsally dark brown to brown, but mesoscutum yellowish, lighter than head in D. (S.) verae sp. nov., Figs 16, 99), T1 strigose (rugose–foveolate in D. (S.) verae sp. nov., Figs 21, 104), fore wing vein (r+3-SR) 5.6–5.9× as long as r-m (4.8–5.1× in D. (S.) verae sp. nov., Figs 18, 101), hind femur 4.7–5.0× as long as wide (4.2–4.3× in D. (S.) verae sp. nov.), hind tibia 9.8–10.0× as long as wide (8.6–8.9× in D. (S.) verae sp. nov., Figs 20, 105).

#### Description.

♀. Length. Body: 2.5–2.8 mm. Fore wing: 2.6–2.9 mm. Hind wing: 2.0–2.1 mm.

***Head***: in dorsal view (Fig. 16), 1.7–1.9× as wide as long, 1.3× as wide as mesosoma, as wide at eyes as temples or slightly wider at eyes. Frons smooth or with weak mid groove. POL 1.5× as OD, OOL 2.6× as OD. In lateral view (Fig. 17), eye 1.4–1.5× as high as wide, 1.1–1.2× as wide as temple. Face 1.6× as wide as high (Fig. 14), 1.8–2.0× as wide as clypeus, smooth or punctate, with a weak longitudinal ridge dorsally. Clypeus 1.8–1.9× as wide as high, concave ventrally. Malar space 0.5–0.6× as clypeus height. Paraclypeal fovea middle size. Mandible 1.2–1.4× as long as wide, diagonal carina present, weak. Mandibular apex 1.2× wide as base. Upper tooth rounded. Middle tooth acute, longer than other teeth. Lower tooth rounded or nearly so, as long as upper tooth. Upper tooth ca as wide as lower, wider than middle tooth. Antenna with 18–20 segments (Fig. 15), 0.7–0.9× as long as body. Scape 1.8× as long as pedicel. F1 2.7–3.0× as long as wide, 1.2× as long as F2. F2 1.9–2.2× as long as wide. F3 1.8–2.0× as long as wide. AF 2.1–2.2× as long as wide. Maxillary palp 1.2× as long as head height.

***Mesosoma***: 1.2–1.4× as long as high (Fig. 17), 2.0–2.1× as long as wide. Mesoscutum as long as wide, notauli absent on horizontal surface of mesoscutum (Fig. 16). Mesoscutal pit present, oval to elongate, occupying 0.1–0.2× of mesoscutal length. Prescutellar depression 2.0–2.2× as long as wide (Fig. 21), with median carina complete, lateral carinae absent or incomplete anteriorly, very short. Side of pronotum crenulate. Precoxal sulcus crenulate medially, not reaching anterior and/or posterior margins of mesopleuron (Fig. 17). Posterior mesopleural furrow smooth. Propodeum mainly rugulose to rugose (Fig. 21), with areola 0.8× as high as wide; median longitudinal carina complete or nearly so (interrupted very briefly apically); transverse carinae complete. Propodeum with protuberance in lateral view. Propodeal spiracle small to middle (Fig. 17), 0.3–0.4× distance from spiracle to base of propodeum.

**Figures 13–22.**
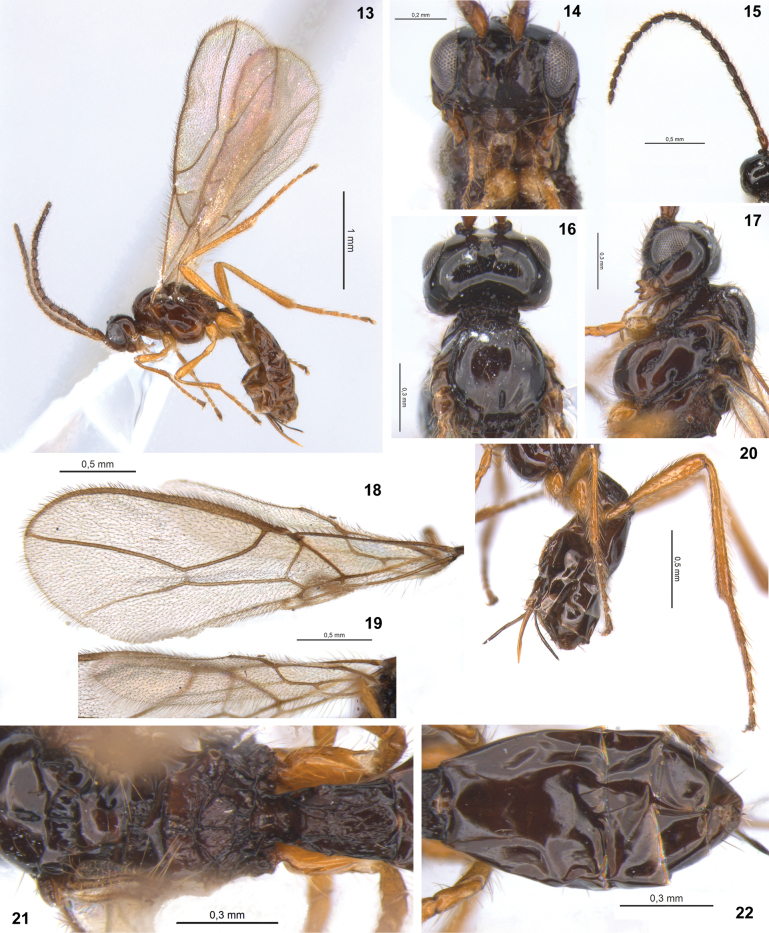
Dinotrema (Synaldis) brunneum sp. nov. (**13** holotype ♀, **14–22** paratype ♀) **13** habitus, lateral view **14** head, frontal view **15** antenna **16** head and mesoscutum, dorsal view **17** head and mesosoma, lateral view **18** fore wing **19** hind wing **20** hind leg, metasoma and ovipositor, lateral view **21** prescutellar depression, propodeum and T1, dorsal view **22** metasoma without T1, dorsal view.

***Wings***: fore wing 2.8–2.9× as long as wide, vein 1-SR present, (r+3-SR) 5.6–5.9× as long as r-m, SR1 1.8–1.9× as long as (r+3-SR); cu-a postfurcal, 1-CU1 0.6–0.7× as long as cu-a. Marginal cell 4.1× as long as wide, submarginal cell 1.9–2.1× as long as wide, first subdiscal cell 2.8–2.9× as long as wide (Fig. 18). Hind wing 5.4× as long as wide, vein 1-M 0.5–0.6× as long as M+CU, 1.3–1.7× as long as 1r-m; m-cu absent (Fig. 19).

***Legs***: hind femur 4.7–5.0× as long as wide. Hind tibia 9.8–10.0× as long as wide, 1.1–1.2× as long as hind tarsus. First segment of hind tarsus 1.8–1.9× as long as second segment (Fig. 20).

***Metasoma***: 1.5–1.7× as long, and as wide as mesosoma (Figs 13, 22). T1 strigose (Fig. 21), 1.7–1.8× as long as wide, apex 1.3–1.6× as wide as base. Ovipositor 0.2–0.3× as long as metasoma, 0.9–1.4× as long as T1, 0.6–0.8× as long as hind femur. Ovipositor sheath with some delicate setae (except on 1/3 apical almost glabrous), 0.2–0.3× as long as metasoma, 0.9–1.2× as long as T1 (Figs 13, 20).

***Color***: dark brown to brown, except mandibles, legs, and ovipositor yellowish. Wigs hyaline, veins brown.

**Male.** Body length 2.9 mm, fore wing 3.1 mm, hind wing 2.2 mm. POL 1.4× as OD, OOL 2.3× as OD. Eye 1.3× as wide as temple. Face 1.45× as long as high. Mandibular apex 1.1× as wide as base. Antenna with 25 segments, 1.1× as long as body. F1 3.3× as long as wide, 1.4× as long as F2. F3 2.3× as long as wide. AF 2.5× as long as wide. Propodeum rugose medially. Fore wing 3.1× as long as wide, vein 1-CU1 0.9× as long as cu-a. First subdiscal cell 2.6× as long as wide. Hind tibia 10.3× as long as wide.

#### Etymology.

The epithet is an adjective derived from *brunneus*, which means brown in Latin. The species name refers to its predominantly brown body color (Figs 13–22).

#### Distribution.

Chile.

### ﻿ Dinotrema (Synaldis) chilense sp. nov.

Taxon classificationAnimaliaHymenopteraBraconidae

B35B7FA2-D9C5-54AD-8633-64D5C6215725

https://zoobank.org/6C3824F7-549C-47CE-B07E-9D69338D71F5

Figs 23–33

#### Type material.

***Holotype***: Chile • ♀ (MNNC); Osorno, Parque Nacional Puyehue, Antillanca; 40°44’S, 72°19’W; alt. 440 m; 14 Jan.–3 Feb. 2017; D. Amorim and V. Silva leg.; sweeping. ***Paratypes***: Chile • 3♀♀ (MNNC); same data as for holotype • 3♀♀ (MNNC) and 1♂ (MNNC); same data as for holotype, except 40°44'06"S, 72°19'47"W; alt. 528 m; flight intercept trap • 1♀ (DCBU 387163) and 1♂ (DCBU 386948); same data as for holotype • 4♀♀ (DCBU 385728, DCBU 386175, DCBU 386465, DCBU 386544); same data as for holotype, except 40°44'06"S, 72°19'47"W; alt. 528 m; flight intercept trap • 1♀ (DCBU 386168); same data as for holotype, except 40°44'06"S, 72°19'47"W; alt. 528 m; Malaise trap.

#### Diagnosis.

This species differs from other New World species of *Synaldis* by the combination of the following characteristics: in lateral view, eye as wide as or slightly wider than temple (Fig. 31); mandible with three relatively large teeth, diagonal carina present, mandibular apex wider than base (Fig. 28); F1 2.8–3.2× as long as wide (Fig. 25); mesoscutal pit present, conspicuous; propodeum with areola, median longitudinal carina incomplete apically, transverse carinae complete (Fig. 27); fore wing vein cu-a distinctly postfurcal, 1-CU1 as long as or longer than cu-a (Fig. 29); hind tibia 9.0–9.4× as long as wide (Fig. 32).

Dinotrema (S.) chilense sp. nov. is similar to D. (S.) brunneum sp. nov. (see their differences in the identification key) and D. (S.) verae sp. nov., differing from the latter by paraclypeal fovea short size (middle in D. (S.) verae sp. nov., Figs 24, 97), fore wing vein (r+3-SR) 5.6–6.4× as long as r-m (5.1× in D. (S.) verae sp. nov.), 1-CU1 1.0–1.4× as long as cu-a (0.55–0.70× in D. (S.) verae sp. nov., Figs 29, 101), hind wing vein 1-M of ♀ 1.7× as long as 1r-m (1.2× in D. (S.) verae sp. nov.), T1 strigose to rugose (rugose–foveolate in D. (S.) verae sp. nov., Figs 27, 104).

Dinotrema (S.) chilense sp. nov. is also somewhat similar to D. (S.) flavum sp. nov., from which it can be distinguished by face and clypeus brown to dark brown (yellow in D. (S.) flavum sp. nov.), AF 2.0–2.3× as long as wide (2.7× in D. (S.) flavum sp. nov.), metasoma 0.8× as wide as mesosoma (1.3× in D. (S.) flavum sp. nov.), hind wing 5.6–5.8× as long as wide and vein 1-M 1.4–1.7× as long as 1r-m (6.2× and 2.0× respectively in D. (S.) flavum sp. nov.), hind femur 4.5–4.9× as long as wide (4.2× in D. (S.) flavum sp. nov.), hind tibia 9.0–9.4× as long as wide (10.1–10.4×in D. (S.) flavum sp. nov., Figs 32, 54).

#### Description.

♀. Length. Body: 1.9–2.6 mm. Fore wing: 2.05–2.65 mm. Hind wing: 1.5–2.0 mm.

***Head***: in dorsal view, 1.7–2.0× as wide as long, 1.3–1.5× as wide as mesosoma, as wide at eyes as at temples or slightly wider at temples. Frons smooth or with weak mid groove. POL 1.2–1.4× as OD, OOL 2.6–3.0× as OD. In lateral view (Fig. 31), eye 1.2–1.5× as high as wide, 1.0–1.2× as wide as temple. Face 1.4–1.7× as wide as high (Fig. 24), 1.8–2.0× as wide as clypeus, smooth or with a weak longitudinal ridge dorsally. Clypeus 1.8–2.1× as wide as high, slightly concave ventrally. Malar space 0.5–0.6× as clypeus height. Paraclypeal fovea short size. Mandible 1.2–1.4× as long as wide (Fig. 28), diagonal carina present. Mandibular apex 1.2–1.4× wide as base. Upper tooth almost rounded. Middle tooth acute, longer than other teeth. Lower tooth rounded, as long as or slightly longer than upper tooth. Upper tooth ca as wide as lower, wider than middle tooth. Antenna with 17–21 segments, 0.9–1.0× as long as body. Scape 1.80–2.05× as long as pedicel. F1 2.8–3.2× as long as wide (Fig. 25), 1.0–1.2× as long as F2. F2 2.2–2.4× as long as wide. F3 1.9–2.2× as long as wide. AF 2.0–2.3× as long as wide (Fig. 26). Maxillary palp 1.1–1.2× as long as head height.

***Mesosoma***: 1.2–1.4× as long as high (Fig. 31), 2.05–2.30× as long as wide. Mesoscutum ca as long as wide, notauli absent on horizontal surface of mesoscutum. Mesoscutal pit present, oval–elongate, occupying 0.1–0.3× of mesoscutal length. Prescutellar depression 2.0–2.2× as long as wide, with median carina complete or incomplete anteriorly (very weak), lateral carinae absent to almost complete. Side of pronotum crenulate, sometimes weakly. Precoxal sulcus crenulate medially, not reaching margins of mesopleuron, or almost reaching its anterior margin. Posterior mesopleural furrow smooth. Propodeum mainly smooth to rugulose (except inside areola with some rugae) (Fig. 27), with areola 1.0–1.1× as high as wide; median longitudinal carina incomplete apically, not extending inside areola or reaching at most its middle; transverse carinae complete. Propodeum with protuberance in lateral view. Propodeal spiracle small to middle (Fig. 31), 0.3–0.4× distance from spiracle to base of propodeum.

**Figures 23–33.**
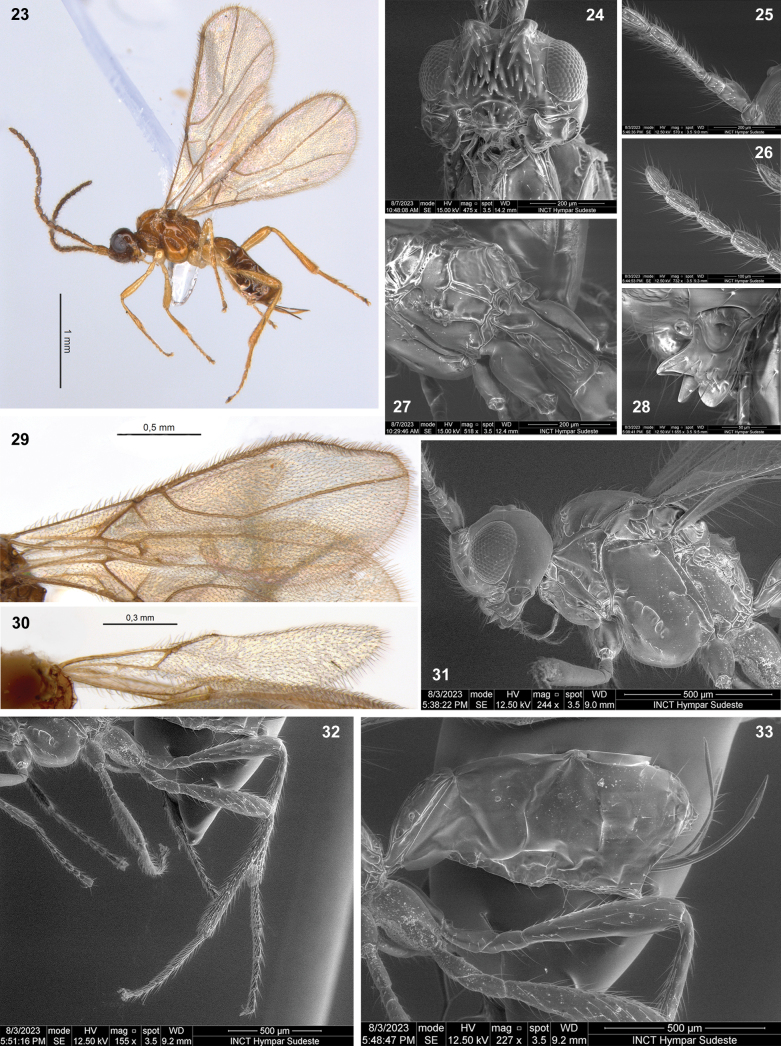
Dinotrema (Synaldis) chilense sp. nov. (**23** holotype ♀, **24–31** paratypes ♀) **23** habitus, lateral view **24** head, frontal view **25, 26** basal and apical parts of antenna respectively **27** propodeum and T1, dorsal view **28** mandible, lateral view **29** fore wing **30** hind wing **31** head and mesosoma, lateral view **32** hind leg **33** metasoma and ovipositor, lateral view.

***Wings***: fore wing 2.6–2.9× as long as wide, vein 1-SR present, (r+3-SR) 5.6–6.4× as long as r-m, SR1 2.0–2.2× as long as (r+3-SR); cu-a distinctly postfurcal, 1-CU1 1.2–1.4× as long as cu-a. Marginal cell 4.4–4.6× as long as wide, submarginal cell 2.2–2.5× as long as wide, first subdiscal cell 3.0–3.3× as long as wide (Fig. 29). Hind wing 5.6–5.8× as long as wide, vein 1-M 0.5–0.6× as long as M+CU, 1.7× as long as 1r-m; m-cu absent (Fig. 30).

***Legs***: hind femur 4.5–4.7× as long as wide. Hind tibia 9.0–9.4× as long as wide, 1.0–1.2× as long as hind tarsus. First segment of hind tarsus 1.9–2.2× as long as second segment (Fig. 32).

***Metasoma***: 1.4–1.8× as long, and 0.8× as wide as mesosoma (Fig. 23). T1 strigose to rugose (Fig. 27), 1.9–2.2× as long as wide, apex 1.2–1.5× as wide as base. Ovipositor 0.35–0.50× as long as metasoma, 1.20–1.75× as long as T1, 0.7–1.2× as long as hind femur. Ovipositor sheath with some delicate setae (except on 1/4 apical almost glabrous), 0.35–0.50× as long as metasoma, 1.20–1.55× as long as T1 (Figs 23, 33).

***Color***: head brown to dark brown. Mesosoma light brown to yellow. Mandibles and legs yellowish. Antennae and metasoma brown to yellow. Wings hyaline to slightly darkened, veins light brown to brown.

#### Variation.

The female of this species has two morphological groups, characterized by:

I) fore wing vein (r+3-SR) 5.5–5.9× as long as r-m, OOL 2.6–2.8× as OD, body length 2.2–2.6 mm,

II) fore wing vein (r+3-SR) 6.3–6.4× as long as r-m, OOL 2.9–3.0× as OD, body length 1.9–2.2 mm.

**Male.** Body length 1.4–2.1 mm, fore wing length 1.5–1.7 mm, hind wing length 1.1–1.7 mm. OOL 2.6–3.4× as OD. Antenna with 18–23 segments, 1.2–1.3× as long as body. F3 2.5× as long as wide. Fore wing vein SR1 2.4× as long as (r+3-SR), marginal cell 4.2× as long as wide. Hind wing vein 1-M 1.4–1.7× as long as 1r-m. Metasoma as wide as mesosoma.

#### Etymology.

The name of species *chilense* is a gentilic adjective derived from Latin in reference to Chile, the country where this species was found.

#### Distribution.

Chile.

### ﻿ Dinotrema (Synaldis) daltoni sp. nov.

Taxon classificationAnimaliaHymenopteraBraconidae

1A1709DA-1939-51F7-B6A7-32E506BBC508

https://zoobank.org/F2451134-8798-42F3-AD97-539322A274D8

Figs 34–45

#### Type material.

***Holotype***: Chile • ♀ (MNNC); Osorno, Parque Nacional Puyehue, Antillanca; 40°44'06"S, 72°19'47"W; alt. 528 m; 14 Jan.–3 Feb. 2017; D. Amorim and V. Silva leg.; flight intercept trap. ***Paratypes***: Chile • 1♀ (MNNC); same data as for holotype, except 40°44’S, 72°19’W; alt. 440 m; sweeping • 1♂ (MNNC); same data as for holotype, except 40°46'55"S, 72°12'39"W; alt. 987 m; 23 Dec. 2019–6 Jan. 2020; Malaise trap • 2♀♀ (DCBU 386560, DCBU 386360); same data as for holotype • 1♀ (DCBU 387144); same data as for holotype, except 40°44’S, 72°19’W; alt. 440 m; sweeping.

#### Diagnosis.

This species differs from other New World species of *Synaldis* by the combination of the following characteristics: in lateral view, eye shorter than temple (Fig. 39); mandible with three relatively large teeth, mandibular apex wider than base (Fig. 37); malar space 0.8× as clypeus height (Fig. 36); F1 2.3–2.5× as long as wide (Fig. 38); mesoscutal pit present, although weak (Fig. 41); propodeum with areola, median longitudinal carina incomplete, basal (not extending inside areola), transverse carinae complete (Fig. 42); propodeal spiracle of ♀ large (Fig. 39) and ♂ middle; fore wing of ♀ with vein cu-a almost interstitial to slightly postfurcal, 1-CU1 distinctly shorter than cu-a; hind tibia 8.1–8.4× as long as wide (Fig. 43).

**Figures 34–45.**
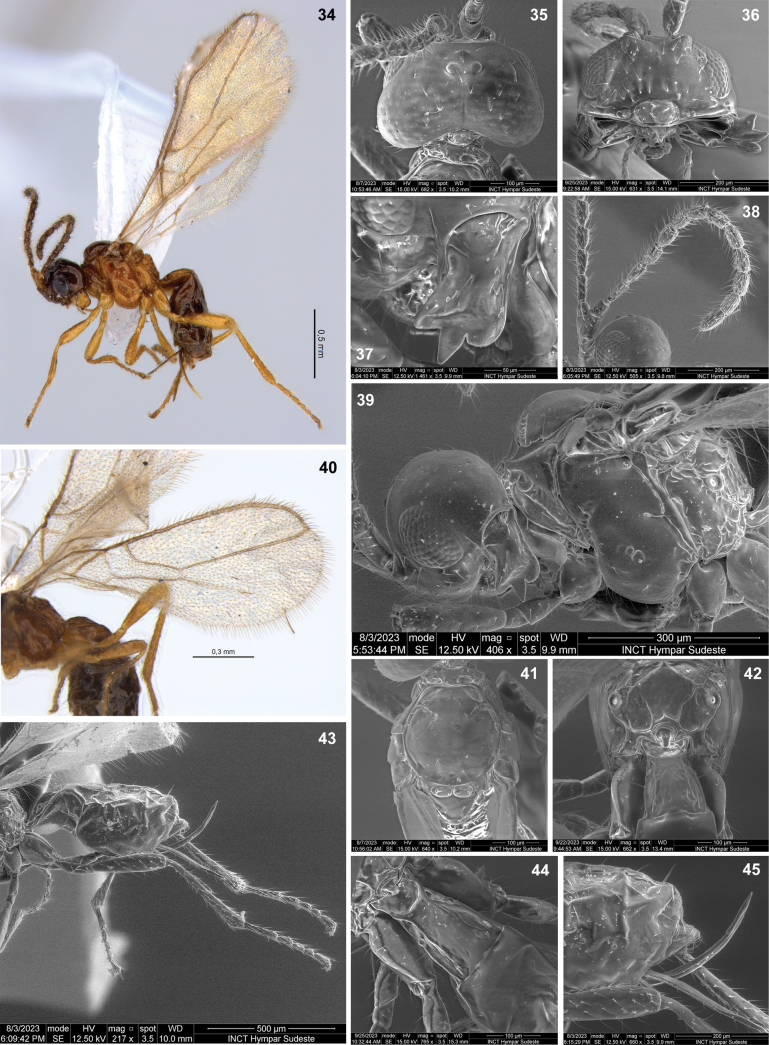
Dinotrema (Synaldis) daltoni sp. nov. (**34** holotype ♀, **35–45** paratypes ♀) **34** habitus, lateral view **35, 36** head, dorsal and frontal view respectively **37** mandible, lateral view **38** antenna **39** head and mesosoma, lateral view **40** fore wing **41** mesonotum, dorsal view **42** propodeum and T1, dorsal view **43** metasoma and hind leg, lateral view **44** anterior part of metasoma, dorsal view **45** apex of metasoma and ovipositor, lateral view.

Dinotrema (S.) daltoni sp. nov. is similar to D. (S.) perisfelipoi sp. nov., from which it can be distinguished by fore wing vein (r+3-SR) 5.0–5.3× as long as r-m (6.2–6.3× in D. (S.) perisfelipoi sp. nov., Figs 34, 69), hind femur 3.7–4.2× as long as wide (4.6–4.8× in D. (S.) perisfelipoi sp. nov.), hind tibia 8.1–8.4× as long as wide (8.9–9.2× in D. (S.) perisfelipoi sp. nov., Figs 43, 71), propodeal spiracle of ♀ large and ♂ middle (♀ middle and ♂ small in D. (S.) perisfelipoi sp. nov., Figs 39, 68). Dinotrema (S.) daltoni sp. nov. is also similar to D. (S.) puyehue sp. nov., their differences are given in the identification key.

#### Description.

♀. Length. Body: 1.5–1.9 mm. Fore wing: 1.5–2.0 mm. Hind wing: 1.05–1.40 mm.

***Head***: in dorsal view (Fig. 35), 1.6–2.0× as wide as long, 1.5–1.6× as wide as mesosoma, wider at temples than eyes. Frons smooth. POL 1.2–1.3× as OD, OOL 3.4× as OD. In lateral view, eye 1.3–1.6× as high as wide, 0.6–0.8× as wide as temple (Fig. 39). Face 1.7–1.9× as wide as high (Fig. 36), 2.1× as wide as clypeus, with longitudinal ridge dorsally. Clypeus 2.0–2.2× as wide as high, straight ventrally. Malar space 0.8× as clypeus height. Paraclypeal fovea short size. Mandible 1.2–1.4× as long as wide, smooth or with diagonal carina weak (Fig. 37). Mandibular apex 1.2–1.3× wide as base. Upper tooth slightly rounded. Middle tooth subacuminate to slightly acute, longer than other teeth. Lower tooth largely rounded, as long as upper or slightly longer than upper tooth. Upper tooth ca as wide as middle, narrower than lower tooth. Antenna with 14–15 segments (Fig. 38), 0.6–0.7× as long as body. Scape 1.9–2.0× as long as pedicel. F1 2.3–2.5× as long as wide, 1.1–1.2× as long as F2. F2 1.80–1.95× as long as wide. F3 1.5–1.6× as long as wide. AF 2.1–2.4× as long as wide. Maxillary palp 0.8–0.9× as long as head height.

***Mesosoma***: 1.2–1.3× as long as high (Fig. 39), 2.1× as long as wide. Mesoscutum ca as long as wide, notauli absent on horizontal surface of mesoscutum (Fig. 41). Mesoscutal pit present, weak, rounded or slightly elongate, occupying 0.05–0.15× of mesoscutal length. Prescutellar depression 2.4–2.5× as long as wide, with median carina complete, smooth laterally. Side of pronotum weakly crenulate. Precoxal sulcus crenulate medially, short, not reaching anterior and/or posterior margins of mesopleuron (Fig. 39). Posterior mesopleural furrow smooth. Propodeum mainly smooth to rugulose (Fig. 42), with areola 1.1× as high as wide; median longitudinal carina incomplete, not extending inside areola; transverse carinae complete. Propodeum without protuberance in lateral view. Propodeal spiracle large, 0.6–0.7× distance from spiracle to base of propodeum (Figs 39, 42).

***Wings***: fore wing 2.8–2.9× as long as wide, vein 1-SR present, (r+3-SR) 5.0–5.3× as long as r-m, SR1 2.4–2.6× as long as (r+3-SR); cu-a almost interstitial or slightly postfurcal, 1-CU1 0.3× as long as cu-a. Marginal cell 5.0× as long as wide, submarginal cell 2.2–2.5× as long as wide, first subdiscal cell 3.1–3.2× as long as wide (Figs 34, 40). Hind wing 5.5× as long as wide, vein 1-M 0.5–0.6× as long as M+CU, 1.5–1.9× as long as 1r-m; m-cu absent.

***Legs***: hind femur 3.7–4.0× as long as wide. Hind tibia 8.1–8.4× as long as wide, 1.1–1.2× as long as hind tarsus. First segment of hind tarsus 1.80–1.95× as long as second segment (Fig. 43).

***Metasoma***: 1.50–1.65× as long, and 1.4× as wide as mesosoma (Fig. 34). T1 smooth to strigose (Fig. 44), 1.7–2.0× as long as wide, apex 1.4–1.5× as wide as base. Ovipositor 0.4–0.8× as long as metasoma, 1.6–2.9× as long as T1, 1.1–1.9× as long as hind femur. Ovipositor sheath with some delicate setae (except on 1/4 apical almost glabrous), 0.4× as long as metasoma, 1.4–1.6× as long as T1 (Figs 43, 45).

***Color***: dark brown to light brown, except mandibles, mesopleuron, propodeum, T1, and legs yellowish. Wings hyaline, veins brown.

**Male.** Face 1.5× as wide as high. Clypeus 1.85× as wide as high, slightly concave ventrally. Mandibular middle tooth acute; upper tooth ca as wide a lower, wider than middle tooth. Antenna with 20 segments, 1.1× as long as body. F1 as long as F2. F3 1.7× as long as wide. Mesosoma 1.9× as long as wide. Propodeal spiracle middle size, 0.5× distance from spiracle to base of propodeum. Fore wing 2.6× as long as wide, vein 1-SR absent, SR1 2.3× as long as (r+3-SR), 1-CU1 0.75× as long as cu-a, submarginal cell 2.05× as long as wide. Hind wing 5.6× as long as wide. Hind femur 4.2× as long as wide. First segment of hind tarsus 2.1× as long as second. Metasoma 1.4× as long as mesosoma. T1 with apex 1.6× as wide as base. Head, flagellum and metasoma from the second tergite brown; mandibles, scape, pedicel dark yellow; mesosoma, legs, and T1 yellow.

#### Etymology.

The species name *daltoni* is a genitive noun, named in honor of Dalton de Souza Amorim, one of the collectors and who supplied the type material for this species.

#### Distribution.

Chile.

#### Comments.

Dinotrema (S.) daltoni sp. nov. has enlarged propodeal spiracles, similar to the Nearctic species Dinotrema (Synaldis) spiraculosa (Fischer, 1967). However, unlike D. (S.) daltoni sp. nov., in D. (S.) spiraculosa the propodeum lacks an areola (despite being sculptured); the eye is as wide as or wider than temple (in lateral view); and the precoxal sulcus sculpture extends to the anterior margin of the mesopleuron (according to Peris-Felipo and Belokobylskij 2017).

### ﻿ Dinotrema (Synaldis) flavum sp. nov.

Taxon classificationAnimaliaHymenopteraBraconidae

F956A98F-9C9E-57D3-9B25-4AD75B4DFD1B

https://zoobank.org/3C4BD845-A600-488C-AFB5-8250BD643F91

Figs 46–54

#### Type material.

***Holotype***: Chile • ♀ (MNNC); Osorno, Parque Nacional Puyehue, Antillanca; 40°44'06"S, 72°18'47"W; alt. 528 m; 14 Jan.–3 Feb. 2017; D. Amorim and V. Silva leg.; flight intercept. ***Paratype***: Chile • 1♀ (DCBU 385798); same data as for holotype, except 40°46'28"S, 72°12'41"W; alt. 1054 m; sweeping.

#### Diagnosis.

**Figures 46–54.**
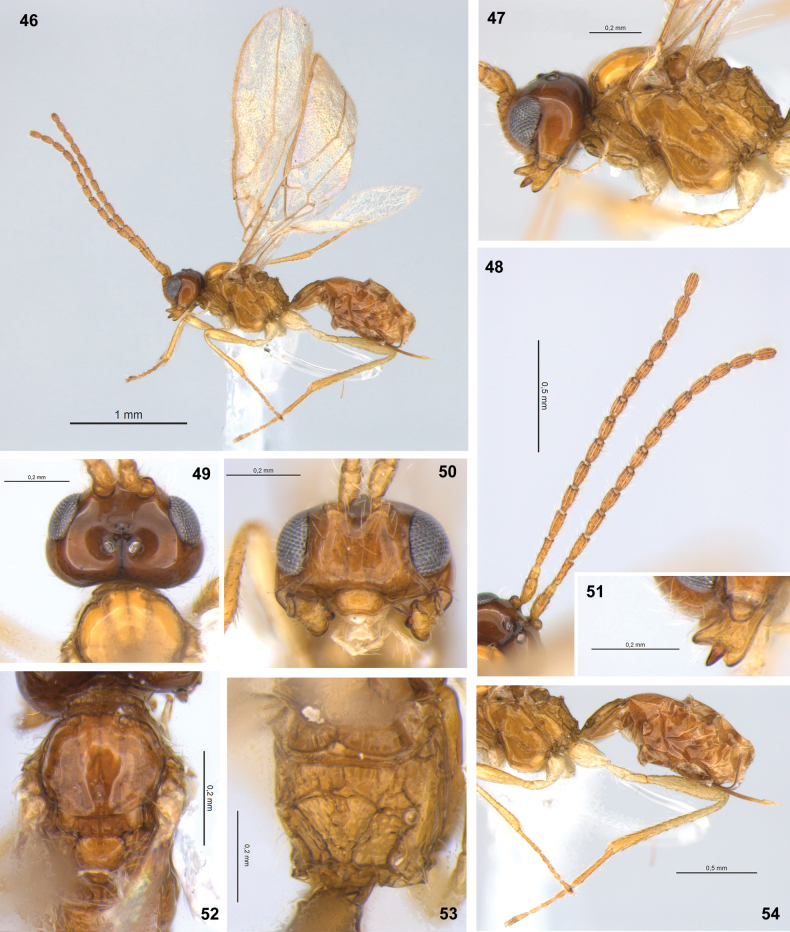
Dinotrema (Synaldis) flavum sp. nov. (holotype ♀, except **52** paratype ♀) **46** habitus, lateral view **47** head and mesosoma, lateral view **48** antenna **49, 50** head, dorsal and frontal view respectively **51** mandible, lateral view **52** mesonotum, dorsal view **53** propodeum, dorsal view **54** hind leg, metasoma and ovipositor, lateral view.

This species differs from other New World species of *Synaldis* by the combination of the following characteristics: face and clypeus yellow (Fig. 50); in lateral view, eye as wide as temple (Fig. 47); mandible with three relatively large teeth, diagonal carina present, mandibular apex wider than base (Fig. 51); F1 2.7–3.1× as long as wide (Fig. 48); mesoscutal pit present, conspicuous (Fig. 52); propodeum with areola, median longitudinal carina incomplete apically, transverse carinae complete (Fig. 53); fore wing vein cu-a distinctly postfurcal, 1-CU1 as long as cu-a (Fig. 46); hind wing vein 1-M 2.0× as long as 1-rm; hind tibia 10.1–10.3× as long as wide (Fig. 54).

Dinotrema (S.) flavum sp. nov. is similar to D. (S.) chilense sp. nov. and D. (S.) puyehue sp. nov. Their distinctions are given, respectively, in the diagnosis of the D. (S.) chilense and identification key.

#### Description.

♀. Length. Body: 1.9–2.4 mm. Fore wing: 2.0–2.4 mm. Hind wing: 1.4–1.7 mm.

***Head***: in dorsal view (Fig. 49), 1.6× as wide as long, 1.50–1.65× as wide as mesosoma, slightly wider at temples than eyes. Frons with weak mid groove. POL 1.1× as OD, OOL 3.0× as OD. In lateral view (Fig. 47), eye 1.4× as high as wide, as wide as temple. Face 1.6× as wide as high (Fig. 50), 1.9–2.0× as wide as clypeus, smooth. Clypeus 2.0–2.1× as wide as high, slightly concave ventrally. Malar space 0.5–0.6× as clypeus height. Paraclypeal fovea short size. Mandible 1.2× as long as wide (Fig. 51), diagonal carina present. Mandibular apex 1.4× wide as base. Upper tooth rounded. Middle tooth acute, longer than other teeth. Lower tooth rounded, as long as upper tooth. Upper tooth ca as wide as lower, wider than middle tooth. Antenna with 18 segments (Fig. 48), as long as body. Scape 1.8–2.0× as long as pedicel. F1 2.7–3.1× as long as wide, 1.1× as long as F2. F2 2.25–2.40× as long as wide. F3 1.8–2.1× as long as wide. AF 2.7× as long as wide. Maxillary palp 1.1× as long as head height.

***Mesosoma***: 1.3× as long as high (Fig. 47), 2.1× as long as wide. Mesoscutum as long as wide, notauli absent on horizontal surface of mesoscutum (Fig. 52). Mesoscutal pit present, oval–elongate, occupying 0.1–0.2× of mesoscutal length. Prescutellar depression 2.5× as long as wide, with median carina complete, lateral carinae almost complete, weak. Side of pronotum crenulate. Precoxal sulcus crenulate medially, not reaching anterior and/or posterior margins of mesopleuron (Fig. 47). Posterior mesopleural furrow smooth. Propodeum mainly rugulose to rugose (Fig. 53), with areola 0.9× as high as wide; median longitudinal carina incomplete, not extending inside areola or reaching its middle at most; transverse carinae complete. Propodeum with protuberance in lateral view. Propodeal spiracle small to middle (Fig. 47), 0.3–0.4× distance from spiracle to base of propodeum.

***Wings***: fore wing 2.7× as long as wide, vein 1-SR present, (r+3-SR) 4.9–5.0× as long as r-m, SR1 2.1–2.3× as long as (r+3-SR); cu-a distinctly postfurcal, 1-CU1 as long as cu-a. Marginal cell 4.5× as long as wide, submarginal cell 2.4× as long as wide, first subdiscal cell 3.0× as long as wide (Fig. 46). Hind wing 6.2× as long as wide, vein 1-M 0.5× as long as M+CU, 2.0× as long as 1r-m; m-cu absent.

***Legs***: hind femur 4.1–4.2× as long as wide. Hind tibia 10.1–10.3× as long as wide, 1.1–1.2× as long as hind tarsus. First segment of hind tarsus 2.2× as long as second segment (Fig. 54).

***Metasoma***: 1.6× as long, and 1.3× as wide as mesosoma (Fig. 46). T1 strigose, 2.15× as long as wide, apex 1.4× as wide as base. Ovipositor 0.4× as long as metasoma, 1.3–1.6× as long as T1, 0.9–1.0× as long as hind femur. Ovipositor sheath with some sparse and delicate setae (except on 1/4 apical almost glabrous), 0.3–0.4× as long as metasoma, 1.20–1.35× as long as T1 (Fig. 54).

***Color***: mainly yellow, except head dorsally brown to light brown; mesosoma dorsally and flagellum yellow to light brown; metasoma from second tergite light brown; ovipositor sheath brown. Wings hyaline, veins light brown.

**Male.** Unknown.

#### Etymology.

The epithet is an adjective derived from *flavus*, which means yellow in Latin. The species name refers to its predominantly yellow body color (Figs 46–54).

#### Distribution.

Chile.

### ﻿ Dinotrema (Synaldis) latusdentertium sp. nov.

Taxon classificationAnimaliaHymenopteraBraconidae

9361A113-2A6B-5C0F-8CF2-F762E0D4FA66

https://zoobank.org/A7DF55C8-8025-4F10-BC68-C5E8D5AC0B3E

Figs 55–64

#### Type material.

***Holotype***: Chile • ♀ (MNNC); Osorno, Parque Nacional Puyehue, Antillanca; 40°46'55"S, 72°12'39"W; alt. 987 m; 16–30 Mar. 2019; D. Amorim and V. Silva leg.; Malaise trap. ***Paratype***: Chile • 1♀ (DCBU 386606); same data as for holotype, except 40°44’S, 72°19’W; alt. 440 m; 14 Jan.–3 Feb. 2017; pan trap.

#### Diagnosis.

This species differs from other New World species of *Synaldis* by the combination of the following characteristics: in lateral view, eye wider than temple, at least slightly (Fig. 60); mandible with three relatively large teeth, diagonal carina present, mandibular apex wider than base (Fig. 57); F1 3.5–3.8× as long as wide (Fig. 59); mesoscutal pit present, conspicuous (Fig. 61); propodeum with areola, median longitudinal carina incomplete to complete, transverse carinae complete (Fig. 62); fore wing vein cu-a distinctly postfurcal, 1-CU1 as long as cu-a (Fig. 63); hind wing 4.9× as long as wide; hind tibia 10.0–10.2× as long as wide (Fig. 64).

Dinotrema (S.) latusdentertium sp. nov. is similar to D. (S.) pilosicaudatum sp. nov., see their distinctions in the identification key.

#### Description.

♀. Length. Body: 2.6–2.8 mm. Fore wing: 3.05–3.30 mm. Hind wing: 2.3–2.4 mm.

***Head***: in dorsal view (Fig. 56), 1.8–1.9× as wide as long, 1.2–1.3× as wide as mesosoma, ca as wide at eyes as temples. Frons smooth or with weak mid groove. POL 1.4–1.6× as OD, OOL 2.6–2.7× as OD. In lateral view (Fig. 60), eye 1.4–1.5× as high as wide, 1.1–1.2× as wide as temple. Face 1.6–1.7× as wide as high (Fig. 58), 1.6–1.9× as wide as clypeus, smooth or with a weak longitudinal ridge dorsally. Clypeus 2.1× as wide as high, almost straight ventrally. Malar space 0.5–0.7× as clypeus height. Paraclypeal fovea short size. Mandible 1.5× as long as wide (Fig. 57), diagonal carina present. Mandibular apex 1.2× wide as base. Upper tooth rounded. Middle tooth subacuminate, longer than other teeth. Lower tooth largely rounded, longer than upper tooth. Upper tooth ca as wide as middle, narrower than lower tooth. Antenna with 20–21 segments (Fig. 59), 0.9× as long as body. Scape 1.5–1.8× as long as pedicel. F1 3.5–3.8× as long as wide, 1.2× as long as F2. F2 2.6–2.8× as long as wide. F3 2.0–2.4× as long as wide. AF 2.4–2.6× as long as wide. Maxillary palp 1.4× as long as head height.

***Mesosoma***: 1.2–1.3× as long as high (Fig. 60), 1.9–2.0× as long as wide. Mesoscutum as long as wide, notauli absent on horizontal surface of mesoscutum (Fig. 61). Mesoscutal pit present, oval–elongate, occupying 0.2× of mesoscutal length. Prescutellar depression 2.1–2.3× as long as wide, with median carina complete, smooth laterally. Side of pronotum crenulate. Precoxal sulcus crenulate medially, not reaching anterior and/or posterior margins of mesopleuron (Fig. 60). Posterior mesopleural furrow smooth. Propodeum mainly rugulose to rugose (Fig. 62), areola 0.9× as high as wide; median longitudinal carina incomplete (interrupted at mid-areola) to complete; transverse carinae complete. Propodeum with protuberance in lateral view. Propodeal spiracle small to middle (Fig. 60), 0.3–0.4× distance from spiracle to base of propodeum.

***Wings***: fore wing 2.7× as long as wide, vein 1-SR present, (r+3-SR) 6.2× as long as r-m, SR1 1.7–2.0× as long as (r+3-SR); cu-a postfurcal, 1-CU1 as long as cu-a. Marginal cell 4.0× as long as wide, submarginal cell 2.0× as long as wide, first subdiscal cell 2.8× as long as wide (Fig. 63). Hind wing 4.9× as long as wide, vein 1-M 0.5× as long as M+CU, 1.2–1.3× as long as 1r-m; m-cu absent.

**Figures 55–64.**
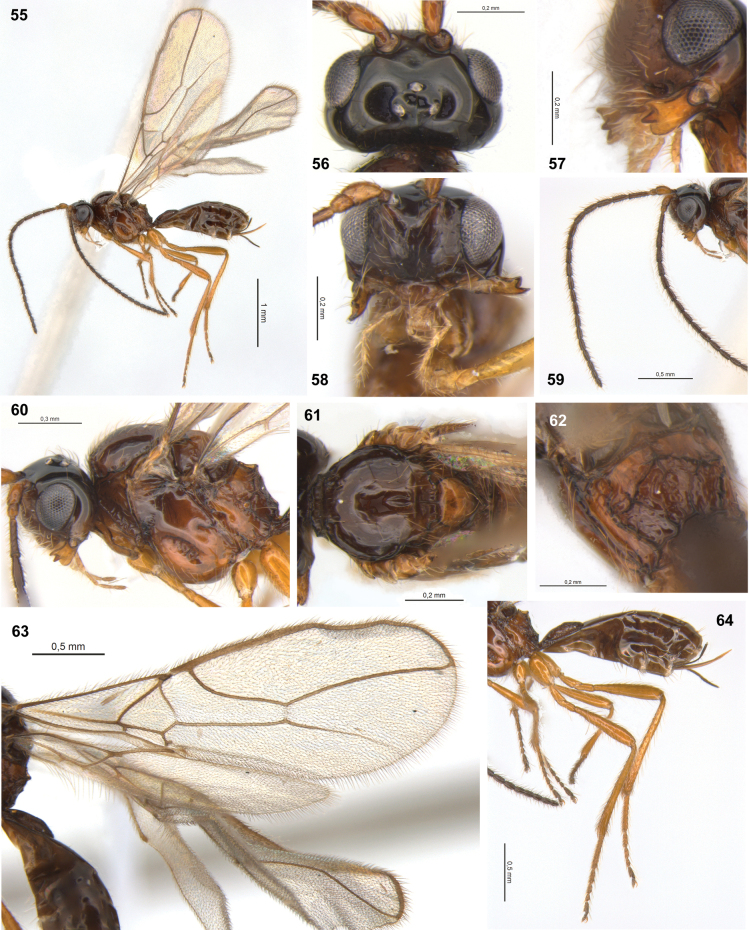
Dinotrema (Synaldis) latusdentertium sp. nov. (holotype ♀, except **58**, **61** paratype ♀) **55** habitus, lateral view **56** head, dorsal view **57** mandible, lateral view **58** head, frontal view **59** antenna **60** head and mesosoma, lateral view **61** mesonotum, dorsal view **62** propodeum, dorsal view **63** wings **64** hind leg, metasoma and ovipositor, lateral view.

***Legs***: hind femur 4.7–5.0× as long as wide. Hind tibia 10.0–10.2× as long as wide, 1.1–1.3× as long as hind tarsus. First segment of hind tarsus 2.0–2.1× as long as second segment (Fig. 64).

***Metasoma***: 1.7× as long, and as wide as mesosoma (Fig. 55). T1 strigose to rugose, 1.70–1.85× as long as wide, apex 1.6× as wide as base. Ovipositor 0.4× as long as metasoma, 1.2–1.5× as long as T1, 0.9× as long as hind femur. Ovipositor sheath with some sparse and delicate setae (except on 1/4 apical almost glabrous), 0.3–0.4× as long as metasoma, 1.1–1.4× as long as T1 (Fig. 64).

***Color***: Head brown to dark brown, except mandibles yellow, scape and pedicel brown to yellowish. Mesosoma brown to light brown, except scutellar disc and tegulae brown to yellow; propleuron, mesopleuron and propodeum orange to yellowish. Legs yellow. Metasoma brown except ovipositor yellow. Wings hyaline, veins brown.

**Male**. Unknown.

#### Etymology.

The epithet is an adjective derived from Latin, combining *latus* (wide), *den* (from *dens*, Latin for tooth), and *tertius* (third). The species name refers to its lower mandibular tooth wider than upper tooth (Fig. 57).

#### Distribution.

Chile.

#### Comments.

Based on the shape of the mandibles, relative length of the flagellar segments, and the propodeal sculpture, D. (S.) latusdentertium sp. nov. can be associated with the Nearctic species Dinotrema (Synaldis) glabrifovea (Fischer, 1967). However, in D. (S.) glabrifovea the mesoscutal pit is absent, the face and clypeus are relatively wider, and the antenna has 25 segments, among other differences (according to Peris-Felipo and Belokobylskij 2017).

### ﻿ Dinotrema (Synaldis) perisfelipoi sp. nov.

Taxon classificationAnimaliaHymenopteraBraconidae

07530056-C9FE-53C2-8363-0344769A0295

https://zoobank.org/3123A760-770C-4AAB-BFFE-34634389F62B

Figs 65–71

#### Type material.

***Holotype***: Chile • ♀ (MNNC); Osorno, Parque Nacional Puyehue, Antillanca; 40°46'55"S, 72°12'39"W; alt. 987 m; 16–30 Mar. 2020; D. Amorim and V. Silva leg.; Malaise trap. ***Paratypes***: Chile • 1♀ (MNNC); same data as for holotype, except 40°44'06"S, 72°19'47"W; alt. 528 m; 14 Jan.–3 Feb. 2017; flight intercept trap • 1♂ (MNNC); same data as for holotype, except 11–25 May. 2019 • 1♀ (DCBU 514624); same data as for holotype • 1 ♀ (DCBU 509530); same data as for holotype, except 9–23 Dec. 2019.

#### Diagnosis.

This species differs from other New World species of *Synaldis* by the combination of the following characteristics: in lateral view, eye shorter than temple (Fig. 66); mandible with three relatively large teeth, diagonal carina present in ♀ (absent in ♂), mandibular apex wider than base; F1 2.4–2.8× as long as wide (Fig. 67); mesoscutal pit present, conspicuous; propodeum with areola, median longitudinal carina incomplete to almost complete, transverse carinae complete (Fig. 70); fore wing vein (r+3-SR) 6.2–6.3× as long as r-m, cu-a postfurcal, 1-CU1 shorter than cu-a (Fig. 69); hind femur 4.6× as long as wide, hind tibia 8.9–9.2× as long as wide (Fig. 71).

Dinotrema (S.) perisfelipoi sp. nov. is similar to D. (S.) daltoni sp. nov. (see their distinctions in the diagnosis of the latter) and D. (S.) puyehue sp. nov., from which it differs by prescutellar depression smooth laterally (with complete lateral carinae in D. (S.) puyehue sp. nov., Fig. 91), fore wing vein (r+3-SR) 6.2–6.3× as long as r-m (5.0–5.2× in D. (S.) puyehue sp. nov.), submarginal cell 2.7–2.9× as long as wide (2.05–2.20× in D. (S.) puyehue sp. nov., Figs 69, 90), AF 2.5–2.6× as long as wide (1.9–2.2× in D. (S.) puyehue sp. nov., Figs 67, 86).

**Figures 65–71.**
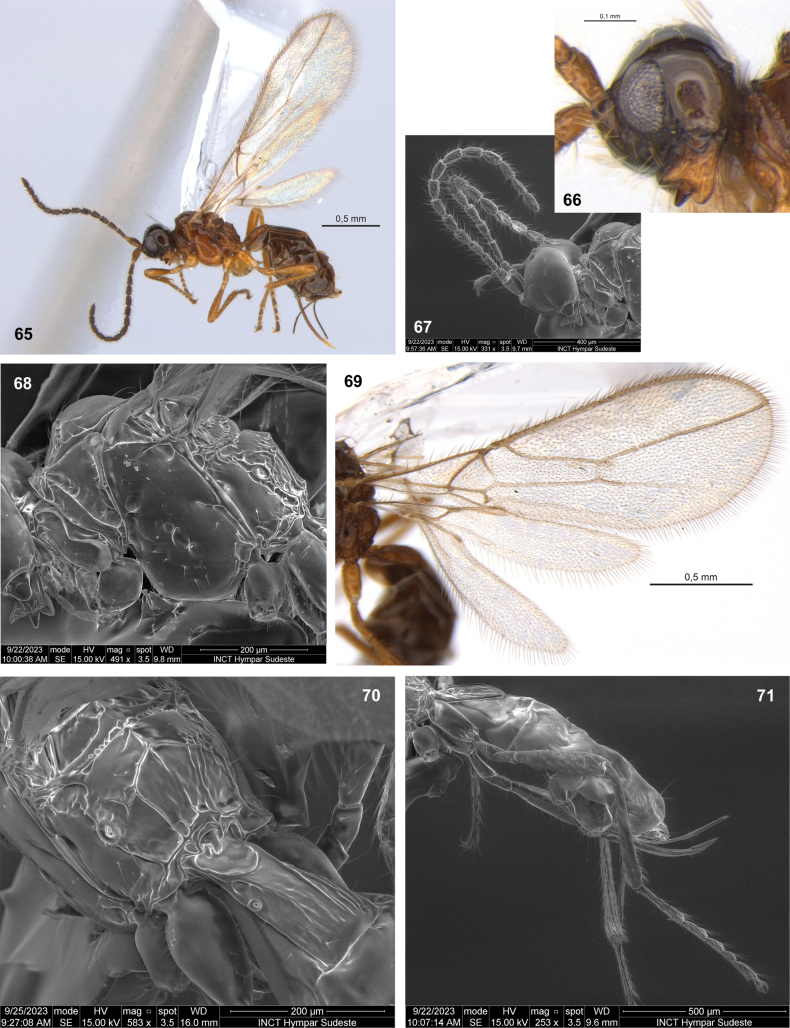
Dinotrema (Synaldis) perisfelipoi sp. nov. (**65, 69** holotype ♀, remainder paratype ♀) **65** habitus, lateral view **66** head and mandible, lateral view **67** antenna **68** mesosoma, lateral view **69** wings **70** propodeum and T1, dorsal view **71** hind leg, metasoma and ovipositor, lateral view.

#### Description.

♀. Length. Body: 1.7–2.1 mm. Fore wing: 1.8–2.1 mm. Hind wing: 1.2–1.4 mm.

***Head***: in dorsal view, 1.7× as wide as long, 1.4× as wide as mesosoma, slightly wider at temples than eyes. Frons smooth. POL 1.30–1.45× as OD, OOL 3.2–3.4× as OD. In lateral view (Fig. 66), eye 1.4× as high as wide, 0.7–0.8× as wide as temple. Face 1.7–2.0× as wide as high, 2.0–2.1× as wide as clypeus, smooth or with a weak longitudinal ridge dorsally. Clypeus 1.7–2.0× as wide as high, concave ventrally. Malar space 0.7× as clypeus height. Paraclypeal fovea short size. Mandible 1.2–1.3× as long as wide (Fig. 66), diagonal carina present. Mandibular apex 1.2–1.3× wide as base. Upper rounded or nearly so. Middle tooth subacuminate, longer than other teeth. Lower tooth largely rounded. Upper tooth ca as wide as middle, narrower than lower tooth. Antenna with 15 segments (Fig. 67), 0.6–0.7× as long as body. Scape 1.9–2.1× as long as pedicel. F1 2.4–2.7× as long as wide, 1.0–1.1× as long as F2. F2 2.0–2.3× as long as wide. F3 1.7–2.2× as long as wide. AF 2.5–2.6× as long as wide. Maxillary palp 0.90–1.05× as long as head height.

***Mesosoma***: 1.2–1.4× as long as high (Fig. 68), 2.1–2.2× as long as wide. Mesoscutum as long as wide, notauli absent on horizontal surface of mesoscutum. Mesoscutal pit present, oval–elongate, occupying 0.1× of mesoscutal length. Prescutellar depression 2.7× as long as wide, with median carina complete (sometimes weak), smooth laterally. Side of pronotum weakly crenulate. Precoxal sulcus crenulate medially, not reaching anterior and/or posterior margins of mesopleuron. Posterior mesopleural furrow smooth. Propodeum mainly smooth to rugulose (Fig. 70), with areola 0.9–1.1× as high as wide; median longitudinal carina incomplete (not extending inside areola) or almost complete (interrupted briefly in mid-areola); transverse carinae complete. Propodeum with very weak protuberance in lateral view. Propodeal spiracle middle (Fig. 68), 0.4–0.5× distance from spiracle to base of propodeum.

***Wings***: fore wing 2.8–2.9× as wide, vein 1-SR absent or present, (r+3-SR) 6.2–6.3× as long as r-m, SR1 2.0–2.5× as long as (r+3-SR); cu-a postfurcal, 1-CU1 0.4–0.7× as long as cu-a. Marginal cell 4.45–4.90× as long as wide, submarginal cell 2.7–2.8× as long as wide, first subdiscal cell 3.1–3.2× as long as wide (Fig. 69). Hind wing 5.35–5.50× as long as wide, vein 1-M 0.5–0.6× as long as M+CU, 1.50–1.85× as long as 1r-m; m-cu absent.

***Legs***: hind femur 4.6× as long as wide. Hind tibia 8.9–9.1× as long as wide, 1.0–1.1× as long as hind tarsus. First segment of hind tarsus 2.0–2.1× as long as second segment (Fig. 71).

***Metasoma***: 1.7–1.9× as long, and 1.4× as wide as mesosoma (Fig. 65). T1 strigose (Fig. 70), 1.4–1.7× as long as wide, apex 1.8× as wide as base. Ovipositor 0.35–0.50× as long as metasoma, 1.5–1.9× as long as T1, 0.9–1.3× as long as hind femur. Ovipositor sheath with some delicate setae (except on 1/4 apical almost glabrous), 0.3–0.4× as long as metasoma, 1.4–1.5× as long as T1 (Fig. 71).

***Color***: brown to yellow, except head dark brown to light brown, and legs entirely yellow or light brown from trochanter. Wings hyaline, veins brown to light brown.

**Male.** Head 1.9× as wide as long. Eye 1.5× as high as wide, 0.85× as wide as temple. Face 2.2× as wide as clypeus. Mandible without diagonal carina. Middle tooth acute. Antenna with 21 segments, 1.2× as long as body. F1 2.8× as long as wide. F2 2.5× as long as wide. F3 2.4× as long as wide. Mesosoma 2.0× as long as wide. Mesoscutal pit occupying 0.2× of mesoscutal length. Prescutellar depression 2.5× as long as wide. Precoxal sulcus almost smooth. Propodeum almost smooth, median longitudinal carina extending to mid-areola. Propodeal spiracle small, 0.25× distance from spiracle to base of propodeum. Fore wing 2.5× as long as wide. Submarginal cell 2.9× as long as wide. Hind femur 4.8× as long as wide. Hind tibia 9.2× as long as wide. Metasoma 1.5× as long as mesosoma. T1 2.4× as long as wide, apex 1.1× as wide as base.

#### Etymology.

The species name *perisfelipoi* is a genitive noun, named in honor of Francisco Javier Peris Felipo, an expert in Alysiinae wasps, who has made significant contributions, particularly in his study of the genus *Dinotrema*.

#### Distribution.

Chile.

### ﻿ Dinotrema (Synaldis) pilosicaudatum sp. nov.

Taxon classificationAnimaliaHymenopteraBraconidae

5E0E9B2F-3CAD-5AC4-BB6B-19C325330251

https://zoobank.org/59D7DEA1-FF88-4DB4-8729-F5173D560940

Figs 72–82

#### Type material.

***Holotype***: Chile • ♀ (MNNC); Osorno, Parque Nacional Puyehue, Antillanca; 40°46'55"S, 72°12'39"W; alt. 987 m; 16–30 Mar. 2020; D. Amorim and V. Silva leg.; Malaise trap. ***Paratypes***: Chile • 1♂ (MNNC); same data as for holotype, except 40°46'28"S, 72°12'41"W; alt. 1054 m; 14 Jan.–3 Feb. 2017; sweeping • 1♀ (DCBU 514534) and 1♂ (DCBU 514551); same data as for holotype.

#### Diagnosis.

This species differs from other New World species of *Synaldis* by the combination of the following characteristics: in lateral view, eye as wider as or slightly wider than temple (Fig. 74); mandible with three relatively large teeth, diagonal carina present, mandibular apex (at least slightly) wider than base (Fig. 74); F1 3.70–3.85× as long as wide (Fig. 76); mesoscutal pit present, conspicuous (Fig. 77); propodeum with areola, median longitudinal carina and transverse carinae complete (Fig. 81); propodeum with a distinct protuberance in lateral view (Fig. 78); fore wing vein cu-a postfurcal, 1-CU1 as long as or shorter than cu-a (Fig. 79); hind tibia 8.9–9.1× as long as wide; T1 rugose–foveolate (Fig. 80); ovipositor sheath with several distinct, erect setae, except on 1/4 apical almost glabrous (Fig. 82).

Dinotrema (S.) pilosicaudatum sp. nov. is similar to D. (S.) latusdentertium sp. nov., their distinctions are given in the identification key.

#### Description.

♀. Length. Body: 2.0–2.2 mm. Fore wing: 2.4 mm. Hind wing: 1.65 mm.

***Head***: in dorsal view (Fig. 73), 1.7–1.8× as wide as long, 1.4× as wide as mesosoma, ca as wide at eyes as temples. Frons with weak mid groove. POL 1.3× as OD, OOL 2.90–3.15× as OD. In lateral view (Fig. 74), eye 1.4–1.5× as high as wide, 1.0–1.1× as wide as temple. Face 1.6× as wide as high (Fig. 75), 2.0× as wide as clypeus, punctate, with longitudinal ridge dorsally. Clypeus 1.65× as wide as high, slightly concave ventrally. Malar space 0.6× as clypeus height. Paraclypeal fovea short size. Mandible 1.5× as long as wide (Fig. 74), diagonal carina present. Mandibular apex 1.1–1.2× wide as base. Upper tooth almost rounded. Middle tooth acute, longer than other teeth. Lower tooth rounded, as long as upper tooth. Upper tooth as wide as lower, wider than middle tooth. Antenna with 18–20 segments (Fig. 76), 0.9–1.0× as long as body. Scape 2.0× as long as pedicel. F1 3.7–3.9× as long as wide, 1.3–1.4× as long as F2. F2 2.6× as long as wide. F3 2.1× as long as wide. AF 2.0–2.1× as long as wide. Maxillary palp 1.2× as long as head height.

***Mesosoma***: 1.2–1.4× as long as high (Fig. 78), 2.1–2.2× as long as wide. Mesoscutum as long as wide, notauli absent on horizontal surface of mesoscutum (Fig. 77). Mesoscutal pit present, oval–elongate, occupying 0.2–0.3× of mesoscutal length. Prescutellar depression 2.0–2.1× as long as wide, with median carina incomplete posteriorly to complete, smooth laterally. Side of pronotum crenulate. Precoxal sulcus crenulate medially, not reaching anterior and/or posterior margins of mesopleuron (Fig. 78). Posterior mesopleural furrow smooth. Propodeum mainly smooth (Fig. 81), with areola as high as wide; median longitudinal carina complete; transverse carinae complete. Propodeum with a distinct protuberance in lateral view (Fig. 78) Propodeal spiracle small, 0.3× distance from spiracle to base of propodeum.

***Wings***: fore wing 2.5–2.6× as long as wide, vein 1-SR present, (r+3-SR) 5.1–5.4× as long as r-m, SR1 2.0–2.2× as long as (r+3-SR); cu-a postfurcal, 1-CU1 0.7–1.0× as long as cu-a. Marginal cell 4.2–4.3× as long as wide, submarginal cell 2.0–2.2× as long as wide, first subdiscal cell 3.05× as long as wide (Fig. 79). Hind wing 5.8× as long as wide, vein 1-M 0.4× as long as M+CU, 1.2× as long as 1r-m; m-cu absent.

***Legs***: hind femur 4.9–5.2× as long as wide. Hind tibia 8.9–9.1× as long as wide, 1.1–1.2× as long as hind tarsus. First segment of hind tarsus 1.9–2.1× as long as second segment.

**Figures 72–82.**
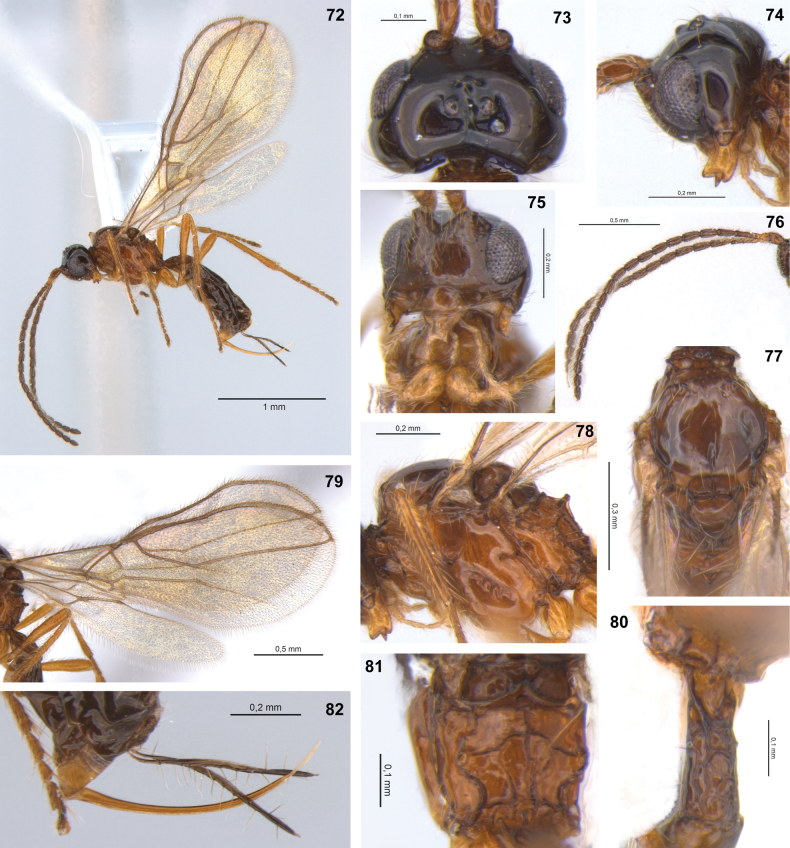
Dinotrema (Synaldis) pilosicaudatum sp. nov. (holotype ♀, except **81**, **82** paratype ♀) **72** habitus, lateral view **73** head, dorsal view **74** head and mandible, lateral view **75** head, frontal view **76** antenna **77** mesonotum, dorsal view **78** mesosoma, lateral view **79** wings **80**T1, dorsal view **81** propodeum, dorsal view **82** ovipositor and sheath, lateral view.

***Metasoma***: 1.6–1.7× as long, and 0.8× as wide as mesosoma (Fig. 72). T1 rugose–foveolate (Fig. 80), 2.1–2.3× as long as wide, apex 1.2–1.3× as wide as base. Ovipositor 0.6–0.7× as long as metasoma, 2.5× as long as T1, 1.5–1.7× as long as hind femur. Ovipositor sheath with several distinct, erect setae (except on 1/4 apical almost glabrous, Fig. 82), 0.5× as long as metasoma, 1.8× as long as T1 (Fig. 72).

***Color***: head dark brown, except mandibles and pedicel yellow. Mesosoma orange–yellow, except pronotum, mesoscutum, scutellum, and metanotum brown. Metasoma brown except ovipositor yellow. Wings hyaline, veins brown.

**Male.** Body length 2.1–2.3 mm. Head 1.3× as wide as mesosoma. POL 1.6× as OD, OOL 2.6× as OD. Face 1.5–1.6× as wide as high, 1.9× as wide as clypeus. Clypeus 1.8× as wide as high. Malar space 0.7× as clypeus height. Mandible 1.4× as long as wide. Antenna with 22 segments, 1.2× as long as body. Scape 1.8× as long as pedicel. F1 1.1–1.3× as long as F2. F2 2.6–2.8× as long as wide. F3 2.3–2.4× as long as wide. AF 2.6× as long as wide. Mesosoma 2.0× as long as wide. Fore wing 2.4× as long as wide, vein SR1 1.8× as long as (r+3-SR). Hind wing 4.65× as long as wide; vein 1-M 0.5× as long as M+CU, 1.3× as long as 1r-m. Hind femur 4.6–5.0× as long as wide. Hind tibia 9.6× as long as wide. Metasoma 1.3–1.5× as long as mesosoma. T1 2.0× as long as wide. Color as in ♀ or brown to dark brown, except mandibles and legs yellow, propleuron, mesopleuron ventrally orange.

#### Etymology.

The epithet is an adjective combining *pilosi* (from *pilosus*, Latin for hairy), *caudatum* (from *cauda*, Latin for tail). The species name refers to its ovipositor sheath with several distinct, erect setae (Fig. 82).

#### Distribution.

Chile.

#### Comments.

Dinotrema (S.) pilosicaudatum sp. nov. can be associated with the Nearctic species D. (S.) glabrifovea (Fischer, 1967) based on similarities in the shape of mandibles, relative length of the flagellomeres, and propodeal sculpture. However, in D. (S.) glabrifovea the mesoscutal pit is absent, the face and clypeus are relatively wider, and the antenna comprises 25 segments, among other distinguishing characteristics (according to Peris-Felipo and Belokobylskij 2017).

### ﻿ Dinotrema (Synaldis) puyehue sp. nov.

Taxon classificationAnimaliaHymenopteraBraconidae

94CBC874-1D43-5D0B-9C8D-B7B5000CC6AB

https://zoobank.org/7B39E2B0-9580-4EA3-8C5F-8279FDDD9696

Figs 83–94

#### Type material.

***Holotype***: Chile • ♀ (MNNC); Osorno, Parque Nacional Puyehue, Antillanca; 40°46'55"S, 72°12'39"W; alt. 987 m; 9–23 Dec. 2019; D. Amorim and V. Silva leg.; Malaise trap. ***Paratypes***: Chile • 1♀ (MNNC) and 1♂ (MNNC); same data as for holotype • 2♀♀ (DCBU 509931, DCBU 514733) and 1♂ (DCBU 509539); same data as for holotype.

#### Diagnosis.

**Figures 83–94.**
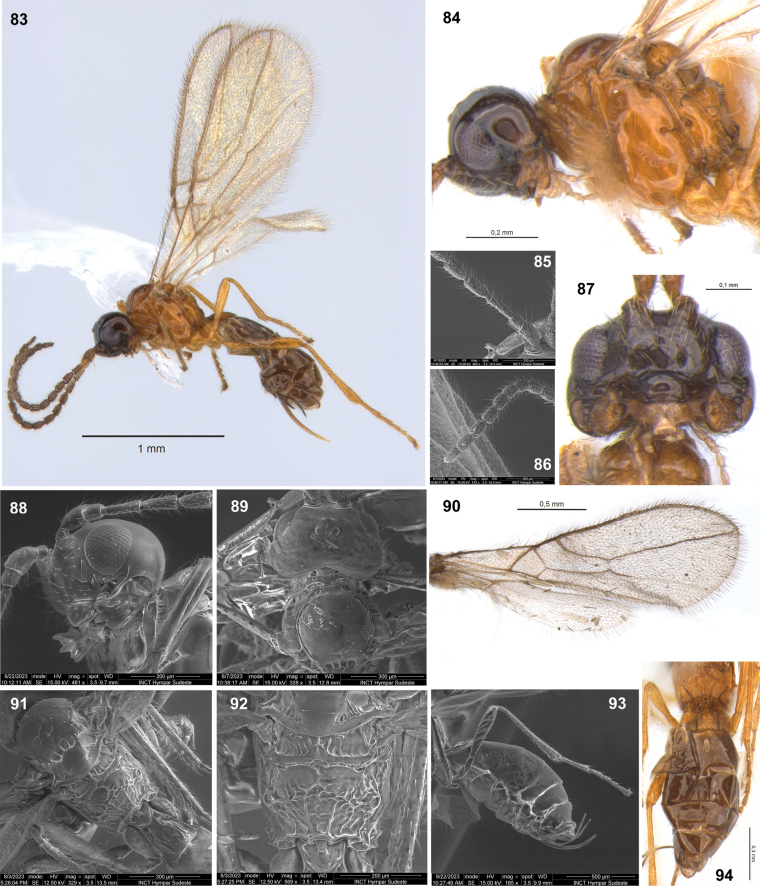
Dinotrema (Synaldis) puyehue sp. nov. (**83**, **84**, **87, 94** holotype ♀, remainder paratype ♀) **83** habitus, lateral view **84** head and mesosoma, lateral view **85, 86** basal and apical parts of antenna respectively **87** head, frontal view **88** mandible, lateral view **89** head and mesoscutum, dorsal view **90** wings **91** mesosoma and T1, dorsal view **92** propodeum, dorsal view **93** hind leg, metasoma and ovipositor, lateral view **94** metasoma, dorsal view.

This species differs from other New World species of *Synaldis* by the combination of the following characteristics: in lateral view, eye shorter than temple, at least slightly (Fig. 84); mandible with three relatively large teeth, diagonal carina present, mandibular apex wider than base (Fig. 88); F1 2.4–2.8× as long as wide (Fig. 85); mesoscutal pit present, conspicuous, prescutellar depression with lateral carinae (Figs 89, 91); propodeum with areola, median longitudinal carina incomplete to complete, transverse carinae complete (Fig. 92); fore wing vein cu-a postfurcal, 1-CU1 shorter than cu-a (Fig. 83); hind tibia 9.4–9.6× as long as wide (Fig. 93); metasoma distinctly wider than mesosoma (Figs 89, 94).

Dinotrema (S.) puyehue sp. nov. is similar to D. (S.) daltoni sp. nov., D. (S.) flavum sp. nov. (their differences are given in the identification key), and D. (S.) perisfelipoi sp. nov. (see their differences in the diagnosis of the latter).

#### Description.

♀. Length. Body: 2.05–2.50 mm. Fore wing: 2.00–2.15 mm. Hind wing: 1.45–1.60 mm.

***Head***: in dorsal view (Fig. 89), 1.6–1.7× as wide as long, 1.5× as wide as mesosoma, as wide at eyes as temples. Frons smooth. POL 1.1–1.2× as OD, OOL 2.7–3.1× as OD. In lateral view (Fig. 84), eye 1.30–1.45× as high as wide, 0.7–0.9× as wide as temple. Face 1.6–1.8× as wide as high (Fig. 87), 1.8–2.0× as wide as clypeus, smooth. Clypeus 2.0–2.1× as wide as high, straight ventrally. Malar space 0.6× as clypeus height. Paraclypeal fovea short size. Mandible 1.2–1.3× as long as wide (Figs 84, 88), diagonal carina present. Mandibular apex 1.2–1.3× wide as base. Upper tooth rounded. Middle tooth subacuminate, longer than other teeth. Lower tooth rounded, as long as upper tooth. Lower tooth as wide as or slightly wider than upper, both wider than middle tooth. Antenna with 16–18 segments (Fig. 83), 0.7–0.8× as long as body. Scape 1.9–2.0× as long as pedicel. F1 2.4–2.6× as long as wide (Fig. 85), 0.95–1.10× as long as F2. F2 2.1–2.3× as long as wide. F3 1.6–1.8× as long as wide. AF 2.0–2.2× as long as wide (Fig. 86). Maxillary palp 0.8–0.9× as long as head height.

***Mesosoma***: 1.1–1.2× as long as high (Fig. 84), 2.1× as long as wide. Mesoscutum as long as wide, notauli absent on horizontal surface of mesoscutum (Fig. 89). Mesoscutal pit present, oval to elongate, occupying 0.1–0.2× of mesoscutal length. Prescutellar depression 2.40–2.65× as long as wide, with median and lateral carinae complete (Figs 89, 91). Side of pronotum almost smooth. Precoxal sulcus crenulate medially, not reaching anterior and/or posterior margins of mesopleuron (Fig. 84). Posterior mesopleural furrow smooth. Propodeum rugulose to rugose (Figs 91, 92), with areola 1.1× as high as wide; median longitudinal carina incomplete (not extending inside areola) to complete; transverse carinae complete. Propodeum with a very weak protuberance in lateral view. Propodeal spiracle middle (Fig. 84), 0.5× distance from spiracle to base of propodeum.

***Wings***: Fore wing 2.6–2.7× as long as wide, vein 1-SR absent or present, (r+3-SR) 5.0–5.1× as long as r-m, SR1 2.3–2.4× as long as (r+3-SR); cu-a postfurcal, 1-CU1 0.4–0.5× as long as cu-a. Marginal cell 4.5–4.9× as long as wide, submarginal cell 2.2× as long as wide, first subdiscal cell 2.9–3.0× as long as wide (Figs 83, 90). Hind wing 5.3× as long as wide, vein 1-M 0.5–0.6× as long as M+CU, 1.9–2.1× as long as 1r-m; m-cu absent.

***Legs***: Hind femur 4.0× as long as wide. Hind tibia 9.4–9.6× as long as wide, 1.1–1.2× as long as hind tarsus. First segment of hind tarsus 1.9–2.1× as long as second segment (Fig. 93).

***Metasoma***: 2.0–2.2× as long, and 1.7× as wide as mesosoma (Figs 83, 89, 94). T1 strigose (Fig. 91), 1.5–1.8× as long as wide, apex 1.45–1.70× as wide as base. Ovipositor 0.4–0.5× as long as metasoma, 1.7–2.1× as long as T1, 0.95–1.40× as long as hind femur. Ovipositor sheath with some sparse and delicate setae (except on 1/3 apical almost glabrous), 0.3× as long as metasoma, 1.3–1.6× as long as T1 (Figs 83, 93).

***Color***: Head brown to dark brown, except mandibles, scape, and pedicel brown to light brown. Mesosoma light brown to yellow. Legs yellow. Metasoma brown, except T1 light brown and ovipositor yellow. Wings hyaline, veins brown.

**Male.** Body length 1.7 mm, fore wing 1.7–1.9 mm, hind wing 1.2–1.3 mm. Face with longitudinal ridge dorsally. Antenna 0.9–1.0× as long as body. F1 2.7–2.8× as long as wide. F2 2.3–2.4× as long as wide. F3 2.1× as long as wide. AF 1.9–2.1× as long as wide. Mesosoma 1.3× as long as high. Propodeal areola as high as wide. Propodeal spiracle small, 0.2× distance from spiracle to base of propodeum. Fore wing vein (r+3-SR) 5.2× as long as r-m, SR1 2.05× as long as (r+3-SR), submarginal cell 2.6× as long as wide. Hind wing 5.5× as long as wide, vein 1-M 1.8× as long as 1r-m. Hind femur 4.2× as long as wide. Hind tibia as long as hind tarsus. Metasoma 1.4× as long as mesosoma.

#### Etymology.

The name of species *puyehue* is a noun in apposition in reference to Parque Nacional de Puyehue, the type locality of the species.

#### Distribution.

Chile.

### ﻿ Dinotrema (Synaldis) verae sp. nov.

Taxon classificationAnimaliaHymenopteraBraconidae

5A2FA59F-EFF9-5014-962F-36A820E43D91

https://zoobank.org/2AB7B5DC-D789-46DE-A12E-8211676F7D3F

Figs 95–105

#### Type material.

***Holotype***: Chile • ♀ (MNNC); Osorno, Parque Nacional Puyehue, Antillanca; 40°44'06"S, 72°18'47"W; alt. 528 m; 14 Jan.–3 Feb. 2017; D. Amorim and V. Silva leg.; flight intercept. ***Paratypes***: Chile • 1♂ (MNNC); same data as for holotype, except 40°44'S, 72°19'W; alt. 440 m; pan trap • 1♀ (DCBU 387214); same data as for holotype, except 40°44'S, 72°19'W; alt. 440 m; sweeping.

#### Diagnosis.

This species differs from other New World species of *Synaldis* by the combination of the following characteristics: in lateral view, eye as wide as or slightly wider than temple (Fig. 96); paraclypeal fovea middle size (Fig. 97); mandible with three relatively large teeth, diagonal carina present, mandibular apex wider than base (Fig. 98); F1 2.7–3.1× as long as wide (Fig. 100); mesoscutal pit present, conspicuous (Fig. 99); propodeum with areola, median longitudinal carina incomplete to complete, transverse carinae complete (Fig. 103); fore wing vein cu-a postfurcal, 1-CU1 shorter than cu-a (Fig. 101); hind tibia 8.6–8.8× as long as wide (Fig. 105); T1 rugose–foveolate (Fig. 104).

Dinotrema (S.) verae sp. nov. is related to D. (S.) brunneum sp. nov. and D. (S.) chilense sp. nov. Their differences are given in the diagnosis of D. (S.) brunneum sp. nov. and D. (S.) chilense sp. nov.

#### Description.

♀. Length. Body: 2.5–2.7 mm. Fore wing: 2.5–2.8 mm. Hind wing: 2.1 mm.

**Figures 95–105.**
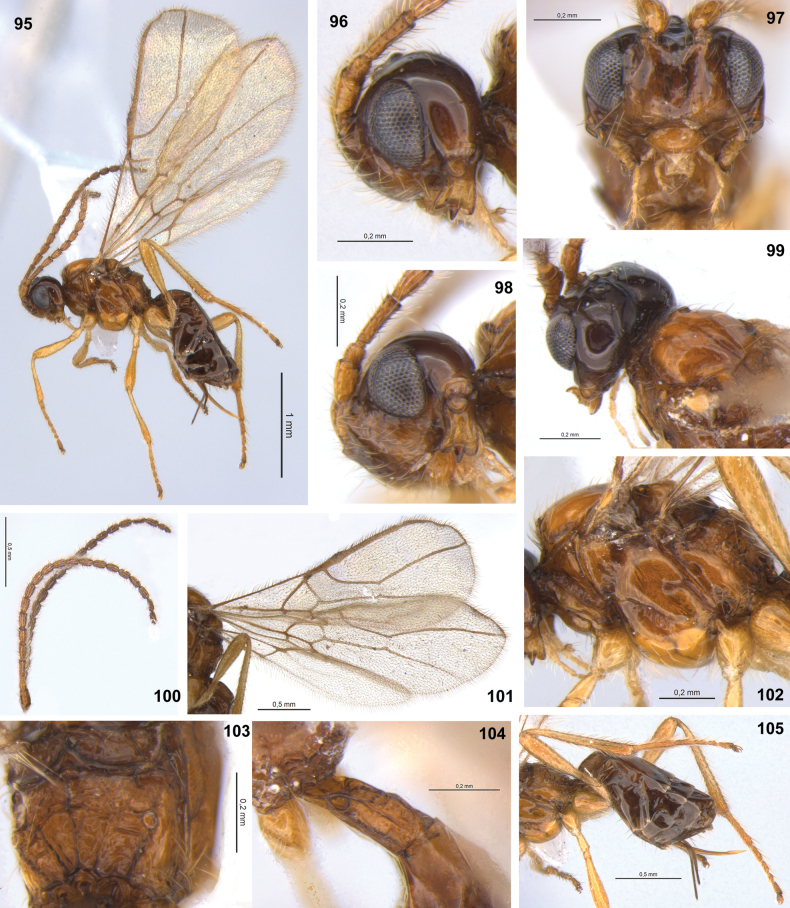
Dinotrema (Synaldis) verae sp. nov. (holotype ♀, except **99**, **100**, **104** paratype ♀) **95** habitus, lateral view **96, 97** head, lateral and frontal view respectively **98** mandible, lateral view **99** head and mesoscutum, dorso-lateral view **100** antennae **101** wings **102** mesosoma, lateral view **103** propodeum, dorsal view **104**T1, dorso-lateral view **105** hind leg, metasoma and ovipositor, lateral view.

***Head***: in dorsal view, 1.6–1.8× as wide as long, 1.3× as wide as mesosoma, slightly wider at temples than eyes. Frons smooth or with weak mid groove. POL 1.2–1.3× as OD, OOL 2.3–2.5× as OD. In lateral view (Fig. 96), eye 1.4–1.5× as high as wide, 1.0–1.1× as wide as temple. Face 1.45–1.60× as wide as high (Fig. 97), 1.9× as wide as clypeus, smooth. Clypeus 1.9× as wide as high, slightly concave ventrally. Malar space 0.6× as clypeus height. Paraclypeal fovea middle size. Mandible 1.2–1.3× as long as wide (Fig. 98), diagonal carina present. Mandibular apex 1.2–1.4× wide as base. Upper tooth rounded. Middle tooth acute, longer than other teeth. Lower tooth rounded, as long as upper tooth. Upper tooth ca as wide as lower, wider than middle tooth. Antenna with 19–20 segments (Fig. 100), 0.8–0.9× as long as body. Scape 2.0× as long as pedicel. F1 2.7–2.8× as long as wide, 1.2× as long as F2. F2 1.8–2.1× as long as wide. F3 1.7–1.8× as long as wide. AF 1.9× as long as wide. Maxillary palp 1.1× as long as head height.

***Mesosoma***: 1.3× as long as high (Fig. 102), 2.0–2.2× as long as wide. Mesoscutum as long as wide, notauli absent on horizontal surface of mesoscutum (Fig. 99). Mesoscutal pit present, oval–elongate, occupying 0.2× of mesoscutal length. Prescutellar depression 2.3–2.4× as long as wide, with median carina complete, smooth laterally. Side of pronotum crenulate. Precoxal sulcus crenulate medially, not reaching anterior and/or posterior margins of mesopleuron (Fig. 102). Posterior mesopleural furrow smooth. Propodeum rugulose to rugose (Fig. 103), with areola 0.8–0.9× as high as wide; median longitudinal carina incomplete (not extending inside areola) to complete; transverse carinae complete. Propodeum with weak protuberance in lateral view. Propodeal spiracle small to middle (Fig. 102), 0.3–0.4× distance from spiracle to base of propodeum.

***Wings***: fore wing 2.8× as long as wide, vein 1-SR present, (r+3-SR) 5.1× as long as r-m, SR1 2.1× as long as (r+3-SR); cu-a postfurcal, 1-CU1 0.55–0.70× as long as cu-a. Marginal cell 4.5× as long as wide, submarginal cell 2.0–2.2× as long as wide, first subdiscal cell 3.1–3.3× as long as wide (Figs 95, 101). Hind wing 5.2× as long as wide, vein 1-M 0.5× as long as M+CU, 1.2× as long as 1r-m; m-cu absent.

***Legs***: Hind femur 4.2–4.3× as long as wide. Hind tibia 8.6–8.8× as long as wide, 1.2× as long as hind tarsus. First segment of hind tarsus 2.0× as long as second segment (Fig. 105).

***Metasoma***: 1.5× as long, and as wide as mesosoma (Fig. 95). T1 rugose–foveolate (Fig. 104), 1.9–2.0× as long as wide, apex 1.5× as wide as base. Ovipositor 0.4× as long as metasoma, 1.45× as long as T1, 0.9–1.0× as long as hind femur. Ovipositor sheath with some delicate setae (except on 1/3 apical almost glabrous), 0.3–0.4× as long as metasoma, 1.10–1.45× as long as T1 (Figs 95, 105).

***Color***: Head dorsally dark brown to brown. Face and clypeus brown to yellowish. Mandibles, antennae, and legs yellow. Mesosoma entirely yellow or parascutellar area and metanotum brown. Metasoma brown to light brown, except T1 brown to yellow and ovipositor yellow. Wings hyaline, veins brown.

**Male.** Body length 2.8 mm. Head 1.45× as wide as mesosoma. Face 1.8× as wide as clypeus. Clypeus 2.0× as wide as high. F1 3.1× as long as wide. F3 2.05× as long as wide. Prescutellar depression with lateral carinae complete, weak. Fore wing vein (r+3-SR) 4.8× as long as r-m, SR1 1.9× as long as (r+3-SR), marginal cell 4.2× as long as wide, first subdiscal cell 2.9× as long as wide. Hind tibia 8.9× as long as wide. Metasoma 2.0× as long as mesosoma. Head brown, except mandibles yellow; mesosoma and metasoma light brown.

#### Etymology.

The species name *verae* is a genitive noun, named after Vera Cristina Silva, one of the collectors of the type material for this species.

#### Distribution.

Chile.

### ﻿Key to the Neotropical species of the subgenus *Synaldis* Foerster, 1863

**Table:** 

1	Propodeum with transverse carinae incomplete, not reaching lateral parts of propodeum (as Fig. 3A, B)	**2**
–	Propodeum with transverse carinae complete, reaching lateral parts of propodeum (as Figs 3C–H, 27, 53, 81)	**3**
2(1)	F1 3.5× as long as wide, 1.3× as long as F2. F2 2.3× as long as wide. Middle flagellomeres 1.8–2.0× as long wide. Propodeal spiracle middle size, 0.4× distance from spiracle to base of propodeum. Hind tibia 7.5× as long as wide. Hind wing 5.7× as long as wide. Antenna with 19–26 segments. Body length 1.8–2.0 mm. Brazil	**D. (Synaldis) novateutoniae (Peris-Felipo, 2017)** ♀♂
–	F1 4.0× as long as wide, ca as long as F2. F2 3.2–3.3× as long as wide. Middle flagellomeres 2.5–2.9× as long as wide. Propodeal spiracle small size, 0.1× distance from spiracle to base of propodeum. Hind tibia 8.2× as long as wide. Hind wing 6.5× as long as wide. Antenna with 18–21 segments. Body length 1.5–1.7 mm. Brazil	**Dinotrema (Synaldis) longiflagellaris (Peris-Felipo, 2017)** ♀♂
3(1)	Propodeum without distinct areola (as Fig. 3C, D). T1 2.7× as long as wide	**4**
–	Propodeum with areola well defined (as Figs 3E–H, 10, 27, 81). T1 1.4–2.4× as long as wide	**5**
4(3)	Face 1.8× as wide as high. Scape 2.5× as long as pedicel. F2 2.2×, and sixth flagellar segment 1.8× as long as wide in ♀. Hind femur 3.5× as long as wide. Hind tibia 8.8× as long as wide. Antenna with 23–25 segments. Body length 2.4–2.7 mm. Brazil	**Dinotrema (Synaldis) fritzi (Peris-Felipo, 2017)** ♀♂
–	Face 1.2× as wide as high. Scape 2.0× as long as pedicel. F2 2.7×, and sixth flagellar segment 2.5× as long as wide. Hind femur 3.9× as long as wide. Hind tibia 9.6× as long as wide. Antenna with 20 segments. Body length 1.7 mm. Brazil	**Dinotrema (Synaldis) magnioculis (Peris-Felipo, 2017)** ♀
5(3)	Mandible not widened towards apex, 1.8× as long as wide. Mandibular teeth relatively small, particularly the upper tooth. Clypeus 3.0× as wide as high. Prescutellar depression 1.1× as long as wide. Eye 1.4× as wide as temple in lateral view. F3 2.7× as long as wide. First segment of hind tarsus 1.5× as long as second segment. Antenna with 20–21 segments. Body length 1.5–1.7 mm. Brazil	**Dinotrema (Synaldis) brasiliense (Peris-Felipo, 2017)** ♀♂
–	Mandible widened to apex (at least weakly), 1.2–1.5× as long as wide. Mandibular teeth relatively large (as Figs 9, 37, 57). Clypeus 1.7–2.2× as wide as high. Prescutellar depression 1.9–2.5× as long as wide. Eye 0.6–1.2× as long as temple in lateral view. F3 1.5–2.4× as long as wide. First segment of hind tarsus 1.8–2.2× as long as second segment	**6**
6(5)	Propodeum with areola but completely lacking a median longitudinal carina (Figs 1E, 10, 11). OOL 4.0× (♀), 3.0× (♂) as OD (Fig. 7). Fore wing with first subdiscal cell 2.5× as long as wide (Fig. 4). Hind wing vein 1-M 2.3–2.4× as long as 1r-m. Antenna with 15–18 segments. Body length 1.6–1.9 mm. Chile (Figs 4–12)	**Dinotrema (Synaldis) acarinareolatum sp. nov.** ♀♂
–	Propodeum with areola and a clear median longitudinal carina, which can be basal (as Fig. 1F) or extend within the areola (as Fig. 1G, H). OOL 2.3–3.4× as OD. Fore wing with first subdiscal cell 2.8–3.3× (♀), 2.3–3.1× (♂) as long as wide. Hind wing vein 1-M 1.2–2.1× as long as 1r-m	**7**
7(6)	Eye 0.6–0.8× as wider as temple in lateral view (as Figs 39, 66)	**8**
–	Eye 0.9–1.2× as wide as temple in lateral view (as Figs 31, 96)	**10**
8(7)	Fore wing vein (r+3-SR) 6.2–6.3× as long as r-m (Fig. 69), submarginal cell 2.7–2.9× as long as wide in ♀, 2.9× in ♂. AF 2.5–2.6× as long as wide (Fig. 67). Hind femur 4.6–4.8× as long as wide (Fig. 71). Antenna with 15 segments in ♀, 21 in ♂. Body length 1.7–2.1 mm. Chile (Figs 65–71)	**Dinotrema (Synaldis) perisfelipoi sp. nov.** ♀♂
–	Fore wing vein (r+3-SR) 5.0–5.3× as long as r-m (Figs 34, 90), submarginal cell 2.2–2.3× as long as wide in ♀, 2.0–2.6× in ♂. AF 1.9–2.3× as long as wide (Figs 38, 86). Hind femur 3.7–4.2× as long as wide (Figs 43, 93)	**9**
9(8)	Prescutellar depression without lateral carinae (Fig. 41). Hind tibia 8.1–8.4× as long as wide (Fig. 43). Metasoma of ♀ 1.50–1.65× as long, and 1.4× as wide as mesosoma (Fig. 34). Propodeal spiracle large size in ♀ and middle in ♂ (0.6–0.7× and 0.5× distance from spiracle to base of propodeum respectively, Fig. 39). Antenna with 14–15 segments in ♀, 20 in ♂. Body length 1.5–1.9 mm. Chile (Figs 34–45)	**Dinotrema (Synaldis) daltoni sp. nov.** ♀♂
–	Prescutellar depression with distinct lateral carinae (Fig. 91). Hind tibia 9.4–9.6× as long as wide (Fig. 93). Metasoma of ♀ 2.0–2.2× as long, and 1.7× as wide as mesosoma (Figs 83, 94). Propodeal spiracle middle size in ♀ and small in ♂ (0.5× and 0.2× distance from spiracle to base of propodeum respectively, Fig. 84). Antenna with 16–18 segments (Fig. 83). Body length 2.05–2.50 mm. Chile (Figs 83–94)	**Dinotrema (Synaldis) puyehue sp. nov.** ♀♂ **(in part 14)**
10(7)	F1 3.5–3.9× as long as wide, F2 2.6–2.8× as long as wide (Figs 59, 76). Mandibular lower tooth wider than upper tooth (Fig. 57) or ovipositor sheath with several distinct erect setae (Fig. 82)	**11**
–	F1 2.4–3.2× as long as wide, F2 1.8–2.4× as long as wide (as Figs 25, 48). Mandibular lower tooth ca as wide as upper tooth (as Figs 28, 51, 88) and ovipositor sheath with some delicate setae (as Fig. 33)	**12**
11(10)	Mandibular lower tooth wider and slightly longer than upper tooth (Fig. 57). Ovipositor sheath with some delicate setae (Fig. 64), 1.1–1.4× as long as T1. Fore wing vein (r+3-SR) 6.2× as long as r-m (Fig. 63). Hind wing 4.9× as long as wide. T1 strigose to rugose, its apex 1.6× as wide as base. Antenna with 20–21 segments. Body length 2.6–2.8 mm. Chile (Figs 55–64)	**Dinotrema (Synaldis) latusdentertium sp. nov.** ♀
–	Mandibular lower tooth ca as wide and as long as upper tooth (Fig. 74). Ovipositor sheath with several distinct and erect setae (Fig. 82), 1.8× as long as T1. Fore wing vein (r+3-SR) 5.1–5.4× as long as r-m (Fig. 79). Hind wing 5.8× as long as wide. T1 rugose–foveolate (Fig. 80), its apex 1.2–1.3× as wide as base. Antenna with 18–22 segments. Body length 2.0–2.3 mm. Chile (Figs 72–80)	**Dinotrema (Synaldis) pilosicaudatum sp. nov.** ♀♂
12(10)	Fore wing vein (r+3-SR) 4.9–5.1× as long as r-m (as Figs 90, 101). Hind femur 4.0–4.3× as long as wide (as Figs 54, 93). Prescutellar depression 2.0–2.2× as long as wide	**13**
–	Fore wing vein (r+3-SR) 5.6–6.4× as long as r-m (Figs 18, 29). Hind femur 4.5–5.0× as long as wide (Figs 20, 32). Prescutellar depression 2.3–2.7× as long as wide	**15**
13(12)	Paraclypeal fovea middle size (Fig. 97, 98). T1 rugose–foveolate (Fig. 104). Hind wing vein 1-M 1.2× as long as 1r-m. Prescutellar depression without lateral carinae (as Fig. 41). Hind tibia 8.6–8.9× as long as wide (Fig. 105). Metasoma as wide as mesosoma. Antenna with 19–20 segments. Body length 2.5–2.8 mm. Chile (Figs 95–105)	**Dinotrema (Synaldis) verae sp. nov.** ♀♂
–	Paraclypeal fovea short size (as Fig. 50). T1 strigose (as Fig. 91). Hind wing vein 1-M 1.8–2.1× as long as 1r-m. Prescutellar depression with lateral carinae, at least incomplete (Figs 52, 91). Hind tibia 9.4–10.3× as long as wide (as Figs 54, 93). Metasoma 1.3–1.7× as wide as mesosoma	**14**
14(13)	Face and clypeus yellow (Fig. 50). Fore wing vein 1-CU1 as long as cu-a (Fig. 46). Metasoma 1.6× as long, and 1.3× as wide as mesosoma. AF 2.7× as long as wide. Hind tibia 10.1–10.3× as long as wide (Fig. 54). Hind wing 6.2× as long as wide. Antenna with 18 segments. Body length 1.9–2.4 mm. Chile (Figs 46–54)	**Dinotrema (Synaldis) flavum sp. nov.** ♀
–	Face and clypeus brown to dark brown (Fig. 87). Fore wing vein 1-CU1 0.4–0.5× as long as cu-a (Fig. 83). Metasoma 2.0–2.2× as long, and 1.7× as wide as mesosoma (Figs 83, 89, 94). AF 1.9–2.2× as long as wide. Hind tibia 9.4–9.6× as long as wide (Fig. 93). Hind wing 5.3–5.5× as long as wide. Antenna with 16–18 segments. Body length 2.05–2.50 mm. Chile (Figs 83–94)	**Dinotrema (Synaldis) puyehue sp. nov.** ♀♂ **(in part 9)**
15(12)	Propodeum brown to dark brown, mainly rugose, with median longitudinal carina complete (or nearly so) (Fig. 21). Paraclypeal fovea middle size (Fig. 14). Fore wing vein 1-CU1 of ♀ 0.6–0.7× as long as cu-a (Fig. 18). Hind tibia 9.8–10.3× as long as wide (Fig. 20). Antenna with 18–20 segments in ♀, 25 in ♂. Body length 1.7–2.8 mm. Chile (Figs 13–22)	**Dinotrema (Synaldis) brunneum sp. nov.** ♀♂
–	Propodeum yellowish, smooth to rugulose, with median longitudinal carina incomplete, clearly lacking apically (Fig. 27). Paraclypeal fovea short size (Fig. 24). Fore wing vein 1-CU1 1.0–1.4× as long as cu-a (Fig. 29). Hind tibia 9.0–9.4× as long as wide (Fig. 32). Antenna with 17–23 segments. Body length 1.4–2.6 mm. Chile (Figs 23–33)	**Dinotrema (Synaldis) chilensis sp. nov.** ♀♂

## ﻿Discussion

The extent and type of sculpture on the propodeum, and the presence/absence of a mesoscutal pit are often used to differentiate species of the subgenera *Dinotrema* and *Synaldis* (Fischer 1967, 1972; Belokobylskij 2004; Tobias 2004a, 2004b; Peris-Felipo et al. 2014a, 2014b; Peris-Felipo and Belokobylskij 2017). In addition, characteristics of mandibles were useful diagnostic characters in the taxonomy of the New World fauna of *Synaldis*. Among the Neotropical species, the development of propodeal transverse carinae and the shape of mandibles allow for the separation of two groups: one containing the species from Brazil, characterized by incomplete propodeal transverse carinae or mandibles with a very small upper tooth (Peris-Felipo and Belokobylskij 2017), and the other consisting of the species from Chile, distinguished by complete propodeal transverse carinae and mandibles with three relatively large teeth. The taxonomic importance of mouth parts in Alysiinae is well-established. Mandibles serve crucial functions in their biology, acting as levers, piercing, or cutting tools for parasitoid to escape from the host puparium and substrate, as well as manipulating substrates during host searching (Griffiths 1964; Wharton 1977, 2017).

On the other hand, most species of *Synaldis* occurring in Nearctic region have mandibles widened towards the apex, with relatively large teeth, like Chilean species (Fischer 1967; Peris-Felipo and Belokobylskij 2017). These Nearctic species exhibit variable propodeal sculpture, but typically lack a distinct areola, differing from Chilean species, which have an areolate propodeum. The exception is D. (S.) glabrifovea. This species has an areolate propodeum, with complete median longitudinal and transverse carinae (as depicted in Fig. 3H). Considering its mandibular shape, propodeal sculpture, and relatively elongated flagellomeres, D. (S.) glabrifovea appears to be related to D. (S.) latusdentertium sp. nov. and D. (S.) pilosicaudatum sp. nov. Nevertheless, D. (S.) glabrifovea differs from the all the Neotropical species of *Synaldis*, including these last ones, by the absence of mesoscutal pit, among other distinguishing characteristics (Peris-Felipo and Belokobylskij 2017).

Differences in propodeal sculpture are commonly used to distinguish species in several genera of Alysiinae. Typically, the propodeum has a median areola with a short median longitudinal carina extending between the areola and its basal margin; however, various transformation series are observed, and in many taxa, the propodeal carination has been completely lost or only a few remnants of it remain. In some groups, the presence of a complete median longitudinal carina appears to result from the gradual narrowing of the areola (Wharton 2002). The ten new species described here have propodea with distinct areolae and complete transverse carinae. In D. (S.) latusdentertium sp. nov., D. (S.) perisfelipoi sp. nov., D. (S.) puyehue sp. nov., and D. (S.) verae sp. nov., the median longitudinal carina varied intraspecifically from incomplete to complete. Therefore, the development of this carina be carefully evaluated at both the specific and interspecific levels. In D. (S.) acarinareolatum sp. nov., the absence of any median longitudinal carina on the areolate propodeum is a notable characteristic.

The relative width of the eye and temple (in lateral view) is another useful character to distinguish some New World species of *Synaldis*. Most of these species have the eye as wide as or wider than temple, while other species have the eye clearly shorter than temple. In a few species, despite that, the temple varies from slightly wider to as wide as eye (Peris-Felipo and Belokobylskij 2017). Variations in wing veins and cells were also significant, especially in fore wing veins 1CU-1 (which affects the position of cu-a in relation to 1-M), 3-SR in relation to r-m, as well as the relative size of the marginal and submarginal cells.

A diagnostic characteristic of the subgenus *Synaldis* as outlined by Peris-Felipo and Belokobylskij (2020), is the consistent postfurcal positioning of the cu-a vein. However, in D. (S.) brunneum sp. nov., D. (S.) daltoni sp. nov., D. (S.) perisfelipoi sp. nov., D. (S.) puyehue sp. nov., and D. (S.) verae sp. nov., the 1-CU1 vein exhibited a degree of reduction relative to cu-a. The most extreme reduction was observed in D. (S.) daltoni sp. nov., with one of the paratypes having the vein 1-CU1 so short that it is difficult to see, rendering the cu-a almost interstitial. The discovery of this condition in this species led to the expansion of the diagnostic criteria for the subgenus.

## Supplementary Material

XML Treatment for Dinotrema

XML Treatment for Subgenus Synaldis

XML Treatment for Dinotrema (Synaldis) acarinareolatum

XML Treatment for Dinotrema (Synaldis) brunneum

XML Treatment for Dinotrema (Synaldis) chilense

XML Treatment for Dinotrema (Synaldis) daltoni

XML Treatment for Dinotrema (Synaldis) flavum

XML Treatment for Dinotrema (Synaldis) latusdentertium

XML Treatment for Dinotrema (Synaldis) perisfelipoi

XML Treatment for Dinotrema (Synaldis) pilosicaudatum

XML Treatment for Dinotrema (Synaldis) puyehue

XML Treatment for Dinotrema (Synaldis) verae

## ﻿Additional information

### Author ORCIDs

Franciélle Dias de Oliveira https://orcid.org/0000-0003-4471-4024

Angélica Maria Penteado-Dias https://orcid.org/0000-0002-8371-5591

### Data availability

All of the data that support the findings of this study are available in the main text.
